# Psychosocial functioning in individuals with advanced oesophago-gastric cancer: a mixed methods systematic review

**DOI:** 10.1186/s12904-023-01288-0

**Published:** 2023-10-28

**Authors:** Cara Ghiglieri, Martin Dempster, Sam Wright, Lisa Graham-Wisener

**Affiliations:** Centre for Improving Health-Related Quality of Life, School of Psychology, Queen’s University Belfast, Belfast, BT7 1NN Northern Ireland

**Keywords:** Oesophago-Gastric Cancer, Gastric Cancer, Oesophageal Cancer, Psychosocial functioning, Health-Related Quality of Life, Palliative Care

## Abstract

**Background:**

Oesophago-gastric cancer is an aggressive disease with a high rate of recurrence and mortality across the disease trajectory. Reduced psychosocial functioning has been evidenced amongst those with advanced disease, however little is known about the contributing factors. Determining these factors is an important clinical consideration to inform assessment and intervention. This review aimed to synthesise the available evidence on the psychosocial functioning of individuals with advanced oesophago-gastric cancer and their carers.

**Methods:**

A JBI mixed-methods systematic review. Four bibliographic databases, MEDLINE, Embase, PsycINFO, and CINAHL, were searched. Quantitative and qualitative studies were screened for inclusion and critically appraised for methodological quality. Both types of data were extracted using JBI tools for mixed-methods systematic reviews. A convergent segregated approach to synthesis and integration was used. The findings of the synthesis have been configured according to JBI methodology.

**Results:**

A total of 12 studies were included in this review, including 6 quantitative studies and 6 qualitative studies. The quantitative results provide preliminary indication of several physical, biological, psychological and macro-level contextual factors associated with psychosocial functioning in this clinical population. The qualitative findings shed light on a range of physical, psychosocial, and existential challenges faced by advanced oesophago-gastric cancer patients. These multiple and often persistent challenges appear to cause considerable distress; however, patients describe the importance of maintaining a sense of normality and control over their illness and its effects. Patients value continuity and structure, however many report shortcomings when accessing care. No findings reporting the experiences from the perspective of carers were found, therefore all findings represent the perspective of the patient.

**Conclusions:**

Further high-quality research is needed to understand how best to support and manage the palliative care needs of individuals living with advanced oesophago-gastric cancer. Implications for practice are discussed, suggesting that psychosocial interventions, complex symptom management and continuity of care could improve the psychosocial functioning of individuals in this setting.

**Pre-registration:**

The systematic review was pre-registered at the International Prospective Register of Systematic Reviews (PROSPERO; CRD42020181273) and the protocol can be viewed on the OSF (http://osf.io/exuzf).

**Supplementary Information:**

The online version contains supplementary material available at 10.1186/s12904-023-01288-0.

## Background

Oesophago-gastric cancer refers to cancer of the oesophagus, stomach, or oesophago-gastric junction. It is a common malignancy, which affects approximately 1.5 million people each year [1], with around 15,000 of those diagnosed in the UK alone [2, 3]. Known for its aggressive nature [4] and high rate of recurrence [5] patients commonly suffer from complex and burdensome symptoms such as dysphagia, fatigue, pain, weight loss, changed bowel habits and psychological distress [6]. Whilst survival rates have improved in the UK in the last 40 years, the majority of patients continue to present with advanced, metastatic disease, thus prognosis remains poor [7]. Consequently, oesophago-gastric cancer represents a major health challenge for patients, carers, the healthcare system and governments alike.

Previous psycho-oncological research has primarily focused on the pre-and post-treatment phases, with particular attention paid to those undergoing treatment with curative intent [8–10]. These studies have highlighted that diagnosis and treatment are associated with impaired psychosocial functioning amongst both patients and their informal carers [8, 11]. For the purpose of this article, we will use the term ‘*psychosocial functioning’* as an umbrella term to refer to how patients and carers function emotionally and socially in daily life following the diagnosis of advanced cancer. In this sense, the term combines psychological functioning, which incorporates, but is not limited to, mood (e.g., depression, anxiety, stress), feelings of self-mastery (e.g. self-efficacy, self-esteem), attributions about responsibility (e.g., guilt, shame), and grief/loss [12], with social functioning, which refers to an individual’s ability to engage effectively in social interactions, to maintain interpersonal relationships, to engage in work, and conduct everyday activities [13]. Ultimately, when combined, the term incorporates well-being, basic functioning, self-mastery and interpersonal and social relationships [14]. In the context of oesophago-gastric cancer, heightened levels of psychological distress have been associated with poorer health-related quality of life outcomes up to two years after surgery [15] and in gastric cancer patients, significantly higher incidence of depressive disorders have been identified compared to their healthy matched cohort [16]. Meanwhile, for oesophageal cancer carers, a longitudinal assessment of distress in longer-term oesophageal cancer carers reported a deterioration from normal to probable anxiety in 35.7% of carers and probable depression in 28.7% carers over time [8]. Whilst these studies have identified that surviving oesophago-gastric cancer and its subsequent treatment can have some adverse consequences for psychosocial functioning of both patients and their carers, there is wider evidence to suggest that palliative care needs increase and quality of life deteriorates once cancer advances beyond a curative state [17]. In the case of advanced oesophago-gastric cancer, the affirmation of irrevocable illness [18], and loss of control over daily life and social relations [19], suggest that the experiences of individuals with advanced oesophago-gastric cancer and their carers will likely differ from those with early, curable disease. Correspondingly, one prospective, longitudinal evaluation of newly diagnosed oesophageal or gastro-oesophageal junction cancer patients, reported that the prevalence of probable depression was greater among patients treated with palliative intent than those treated with curative intent [20].

In recent years, the mainstay of palliative care research has focused on how care should be offered, including optimal models of delivery, timings for referral, and queries regarding who is in greatest need, and whose role it is to provide this care [21]. These questions are particularly pertinent for the advanced oesophago-gastric cancer population, given the scarcity of evidence available to establish which interventions and strategies are most effective in delivering services to advanced oesophago-gastric cancer patients [22] and the limited understanding of the most effective model(s) of care for this clinical population [23].

In the wider literature, several reviews have identified factors associated with psychosocial functioning in advanced cancer patients. For example, high levels of hopelessness, impaired emotional functioning and body image distortions were identified as predictive factors associated with psychological distress in advanced cancer patients [24]. Whilst protective factors such as social support, self-efficacy, emotional distress, and hope have been evidenced to promote psychological resilience [25]. Reviews such as these have provided insight into the complex and varying adjustment processes experienced by advanced cancer patients, however, their focus has been on mixed cancer populations. Whilst commonalities exist between the experience of living with one type of cancer and another, there are unique challenges faced by individuals depending on the location of their cancer, with outcomes varying between cancer type [26]. For example, evidence from a recent systematic review has identified oesophago-gastric cancer patients to be at elevated risk of suicide when compared to other cancer sites [27]. Consequently, it cannot be assumed that the factors influencing psychosocial functioning among individuals with oesophago-gastric cancer and their carers would be the same as those with other advanced cancers. As such, to further develop the evidence base for this clinical population, a comprehensive understanding of the factors associated with psychosocial functioning amongst individuals with advanced oesophago-gastric cancer and their carers, from the perspective of patients, carers and healthcare professionals (HCPs), is needed to inform effective and acceptable palliative care services for this clinical population. Adopting this multi-perspective approach should ensure that knowledge is gained from a triangulation of sources in relation to patients’ and carers’ beliefs, experiences, and needs. In addition, including the perspectives of HCPs should enable the identification of areas in training knowledge needed to improve the support given to patients and carers.

A preliminary search of PROSPERO, MEDLINE and CINAHL was conducted. To date, no previous reviews have systematically examined psychosocial functioning in advanced oesophago-gastric cancer patients and their carers. As such, this mixed-methods review seeks to explore the psychosocial challenges faced by individuals with advanced oesophago-gastric cancer and their carers and identify the factors associated with psychosocial functioning. Findings will be used to assess the current evidence base, recommend areas for future research, and propose clinical recommendations for addressing the psychosocial needs of this clinical population.

### Aim

The aim of this study was to synthesise the available evidence on the psychosocial functioning of individuals with advanced oesophago-gastric cancer patients and their carers.

The research questions were:

I. What are the factors associated with psychosocial functioning in advanced oesophago-gastric cancer patients and their carers?
II. What are the psychosocial challenges faced by individuals living with advanced oesophago-gastric cancer and their carers, from a patient, carer, or HCP perspective?
III. How do patients and their carers experience and adjust to the psychosocial challenges that are initiated from a diagnosis of advanced oesophago-gastric cancer until the end of life, from a patient, carer, or HCP perspective?

## Methods

### Study design

The systematic review was conducted following a convergent segregated approach to synthesis according to the JBI methodology for mixed-methods systematic reviews [28]. This systematic review followed the reporting guidelines of the Preferred Reporting Items for Systematic Reviews and Meta-Analyses Protocols (PRISMA-P) statement [29]. A PRISMA 2020 checklist is included (Supplementary Material 1). The systematic review was pre-registered at the International Prospective Register of Systematic Reviews (PROSPERO; CRD42020181273) and the protocol can be viewed on the OSF (http://osf.io/exuzf).

### Population and settings

The review considered studies that included individuals (≥ 18 years) diagnosed with advanced oesophago-gastric cancer and any close-persons involved in their care, regardless of gender or ethnicity. The review also considered studies that explored the perspectives of HCPs. Studies where the primary tumour site was not in the oesophagus, stomach or oesophago-gastric junction or secondary tumours that have metastasised to this region were excluded. This review considered studies from all geographical regions however as a translation service was not available, only studies published in English were considered for inclusion. It also included all cancer care contexts (e.g., primary care, secondary, tertiary, community or home settings).

### Outcomes and phenomena of interest

The quantitative component of this review considered studies that evaluated psychosocial functioning in advanced oesophago-gastric cancer patients and their carers. In line with the included definition of psycho-social functioning, outcomes and phenomena of interest included domain-specific measures including, but not limited to, anxiety, depression, psychological distress/stress, psychological wellbeing, patient satisfaction, subjective well-being, happiness, existential wellbeing, post-traumatic growth, loneliness, perceptions of social status, subjective wellbeing, and quality of life. For the purpose of this review, the report of a physical symptom alone was not considered to constitute psychosocial functioning, however, where physical functioning was interwoven with psychosocial functioning as part of a Quality of Life (QoL) scale, relevant data was included where possible. The qualitative component of this review considered studies that investigated the psychosocial challenges faced by people diagnosed with advanced oesophago-gastric cancer and their carers and their process of adjustment from a patient, carer, or HCP perspective.

### Types of studies

The review considered empirical quantitative, qualitative or mixed-methods research, all study designs and peer-reviewed publications relevant to the psychosocial functioning of advanced oesophago-gastric patients and their carers. Quantitative studies of an observational nature (e.g., cross-sectional studies, case-control studies, and prospective and retrospective cohort studies) were included. As intervention or treatment efficacy was not being explored, intervention studies were excluded. Qualitative studies including but not limited to phenomenology, grounded theory, narrative analysis, and ethnography were included. Mixed method studies were considered where data from the quantitative or qualitative components could be clearly extracted. Non-empirical research (i.e. systematic reviews, editorials, opinion papers, case studies (case series or case reviews) were excluded as these did not provide sufficient evidence for the associations of interest in this review, however relevant studies were harvested from them, where relevant.

### Search strategy

An initial limited search of Prospero, MEDLINE (Ovid), and PsycInfo (Ovid), was undertaken to identify articles on the topic using the following initial keywords: Oesophageal cancer OR Stomach cancer AND quality of life OR social functioning OR psychological functioning AND advanced OR incurable. The text words contained in the titles and abstracts of relevant articles, and the index terms used to describe the articles were used to develop a full search strategy. The finalised search strategy (Supplementary Material 2) was then applied to the following databases: MEDLINE (Ovid), CINHAL (EBSCO), PsycInfo (Ovid), EMBASE (Ovid). The search strategy, including all identified keywords and index terms was adapted for each included information source. Manual searches were also conducted by examining the reference lists of the included studies and identified reviews and performing citation searches on key articles. Searches were performed on 12th May 2020 and subsequently re-performed on the 6^th of^ July 2022 to include articles published since the initial search until present day. The time frame for the literature search was from database inception to present-day to ensure a comprehensive review of the literature was achieved.

### Quality assessment

Eligible studies were critically appraised for methodological quality by two independent reviewers (CG, SW, LGW) using the JBI Critical Appraisal Checklist Tools in JBI SUMARI [28]. Provided that a study met all of the inclusion criteria, all studies, regardless of the results of their methodological quality, underwent data extraction and synthesis with a view to the contextual information being discussed within the narrative synthesis of the results. Any disagreements that arose between the reviewers were resolved through discussion, or with a third reviewer (LGW).

### Data extraction

Quantitative and qualitative data extraction was completed by two independent reviewers (CG, SW, LGW), using the relevant JBI data extraction tools in JBI SUMARI [28]. The extracted data included the author(s), year of publication, populations, context, geographical location, study methods, outcome measures and findings of significance to the review question. Findings with their corresponding illustrations were then extracted by the primary reviewer (CG) and given a credibility rating. These findings and levels of credibility were then checked for accuracy by a second reviewer (LGW), and any disagreements that arose between the reviewers were resolved through discussion.

### Data synthesis and integration

This review followed a convergent segregated approach to synthesis and integration according to the JBI methodology for mixed-methods systematic reviews using JBI SUMARI [28]. This involved separate quantitative and qualitative synthesis followed by integration of the resultant quantitative evidence and qualitative evidence, which was juxtaposed and organised into a line of argument to produce an overall configured analysis. All authors were involved in reviewing and checking the accuracy of the final findings.

## Results

### Study selection

**Fig. 1:**
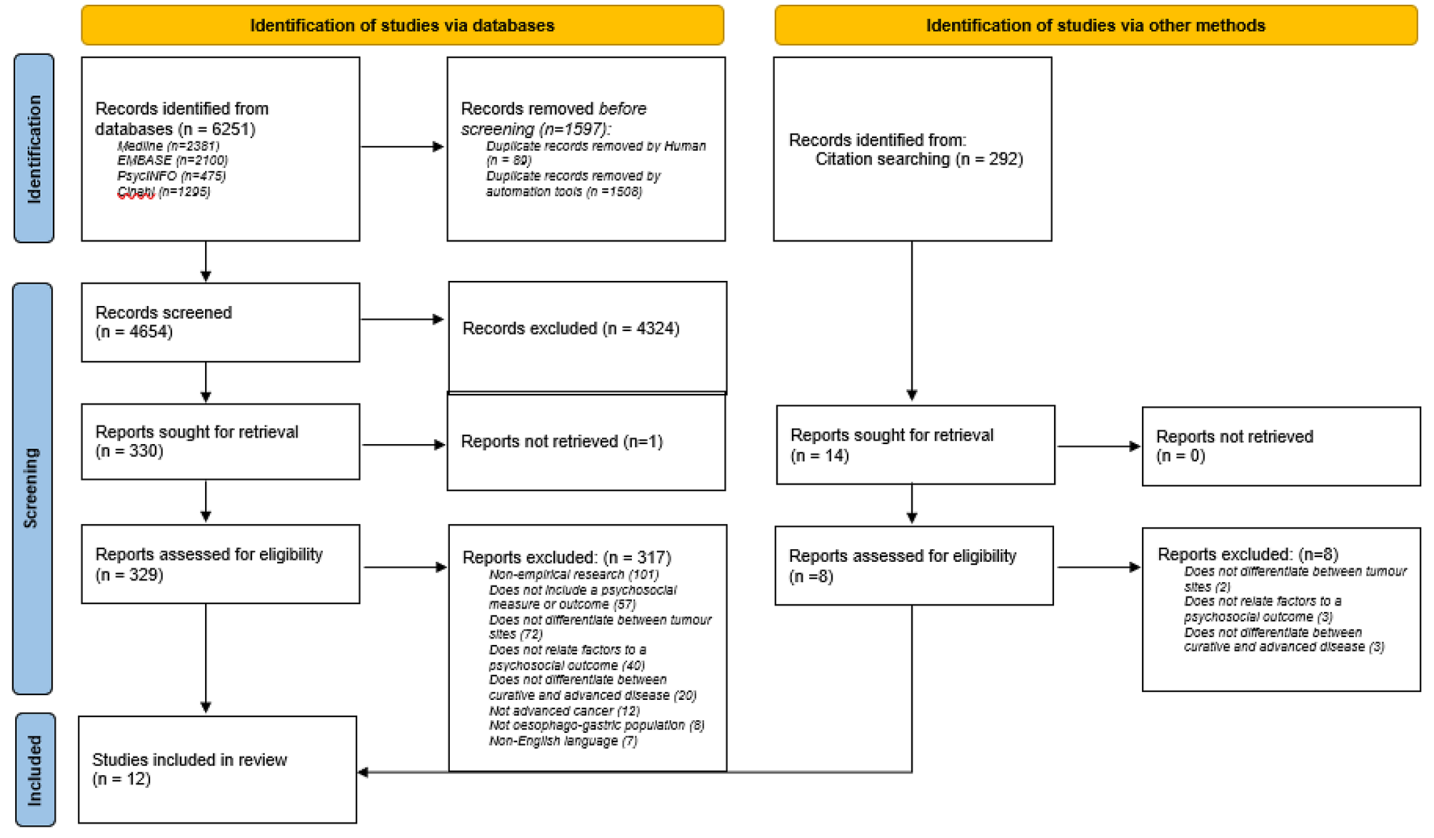
PRISMA 2020 flow diagram for new systematic reviews which included searches of databases and other sources

In total, 6251 articles were identified from the searches, of which 1597 were duplicates and were thus deleted before the remaining 4654 articles were important into Rayyan, a free web and mobile based app for article screening [30]. Each title and abstract were independently screened twice (CG, SW, LGW) following the inclusion and exclusion criteria (above), resulting in the exclusion of 4325 articles. 329 full articles were then retrieved in full and re-assessed twice by independent reviewers (CG, SW, LGW) in detail against the inclusion criteria. Several studies which appeared to meet the inclusion criteria, were excluded at this stage. For example, as treatment efficacy and impact was not being examined, studies where treatment was the only factor measured against quality of life [31–35] were excluded. Studies which measured quality of life but where the outcome was focused on physical functioning without some reference to psychosocial outcomes [36–42] and studies which focused on oesophago-gastric cancers but did not differentiate between curative and advanced disease [43, 44] were excluded. Any disagreements that arose between the reviewers at each stage of the study selection process were resolved through discussion, or with a third reviewer (LGW, MD). As a result, 12 articles were ultimately included in this systematic review. A flowchart presenting the number of studies at each stage of the systematic review and reasons for exclusion is included (Fig. 1).

### Study characteristics

Of the 12 included publications, 6 were quantitative studies and 6 were qualitative studies. The papers were published between 2000 and 2022. The studies were carried out in Denmark [18, 19, 45, 46], Italy [47], South Korea [48, 49], Scotland [50] Japan [51], Canada [52, 53] and one study included pooled global data [54]. Of the included studies, 4 of the papers included advanced gastric cancer patients [45, 46, 48, 50], 6 included advanced oesophageal cancer patients [18, 19, 45–47, 50], 1 included gastric and oesophageal cancer patients [52] and 1 included both gastric and gastroesophageal junction cancer patients [54]. No studies from the perspective of informal carers or HCPs were found regarding the challenges facing patients or informal carers. Details and characteristics of each of the included studies can be seen in the supplementary information (Supplementary Material 3).

### Quality of the included studies

Using JBI critical appraisal checklists in JBI SUMARI [28], appraisal scores were calculated as a proportion of the number of items each study fulfilled on its respective checklist. In general, all studies included in this review were deemed to be of high quality with eleven of the twelve included studies meeting over 80% of the quality criteria. This may have related to the decision to only review published literature, as all studies had been peer reviewed and were therefore considered an acceptable standard prior to publication. The two cross-sectional analytical studies included in this review were considered to be of high-quality, meeting 62.5% and 100% of the criteria. The areas where the Brunelli et al. [47] study did not meet the quality criteria were due to a lack of reporting relating to the inclusion criteria and a lack of reporting relating confounding variables. All four cohort studies met over 90% of the quality appraisal criteria. The areas where the studies did not meet criteria were due to a lack of detailed reporting in relation to the outcomes at the start of the study and details about follow up. In this case, whilst Chau et al. [54] and Koh et al. [48] completed follow ups, neither study described strategies to address incomplete follow-up. As such, this question was marked unclear. In the Bubis et al. [53] study, there were significant differences between the two groups. All other appraisal questions were addressed sufficiently. Finally, all 6 included qualitative studies met between 80 and 95% of the quality appraisal criteria. The reason these studies did not meet certain criteria was again, due to a lack of detailed reporting. Across these studies, only three of the six studies included a statement locating the researcher either culturally or theoretically [19, 45, 46] and similarly, only one reported the role of the researcher in terms of potential impact on the research [50]. Further consideration of the overall methodological quality of included studies has been explored in the individual synthesis of quantitative and qualitative data and the integration of these findings. The checklists for the methodological quality can be found in the supplement information (Supplementary Material 4).

### Description of the quantitative evidence

In this review, six quantitative studies, including 2 cross-sectional analytical studies and 4 cohort studies, provided insight into the factors associated with psychosocial functioning in individuals with advanced oesophago-gastric cancer. The outcomes assessed across the six included studies included Coping Styles to Cancer, Quality of Life, Anxiety, Depression and Overall Well-being. All studies used measures with good evidence for reliability and validity. However, whilst three studies claimed to measure quality of life, Rha et al., [49] measured QoL using the Korean version of the Functional Assessment of Cancer Therapy – Gastric (FACT-Ga) [55] and the Functional Assessment of Chronic Illness Therapy – Spiritual Well-Being (FACIT-Sp) instruments [56] whilst Brunelli et al. [47] and Chau et al. [54] used the The European Organisation for Research and Treatment of Cancer (EORTC) Quality-of-Life Questionnaire (QLQ)-C30). The measures of effect also differed between studies. Where possible, the review authors performed calculations to determine the appropriate effect sizes to report. For the Brunelli et al. [47] and Koh et al. [48] studies, the review authors were able to determine correlation coefficients based on the reported means, SDs and p-values, whereasRha et al., [46] reported these correlation coefficients within their results. In the Chau et al. [54] paper, estimates of odds ratios (ORs) and point change characterising QoL changes from baseline to week 6 as predictors of Performance Status change (a change in the patient’s ability to perform activities of daily living without the help of others) were reported. In the Merchant et al., [52] and Bubis et al. [53] papers, ORs and 95% confidence intervals (Cis) were reported. Due to insufficient homogeneity, statistical pooling was not possible; therefore, a narrative synthesis of the findings was performed. Factors were grouped by type; physical, psychological, biological and macro-level contextual factors. A summary of findings is included in the supplementary information (Supplementary Material 7).

#### Physical factors associated with psychosocial functioning

Of the six included studies, three studies identified a number of physical factors associated with psychosocial functioning in individuals with advanced oesophago-gastric cancer. These included dysphagia [47], proximity to death [53] and performance status [54].

For dysphagia, Brunelli et al. [47] reported that higher grades of dysphagia led to higher levels of QoL impairment in advanced oesophageal cancer patients. The association between dysphagia grade and QoL dimensions proved to be statistically significant for physical functioning (r = 0.34), role functioning (r = 0.3), global QoL (r = 0.29) and fatigue (r = 0.31). Comparison of the health profile of the study sample and a normative sample of cancer patients showed that malignant dysphagia had a relevant impact on all dimensions of QoL considered by the EORTC QLQ C-30 questionnaire [57].

For performance status, Chau et al. [51] reported that positive changes in QoL were directly associated with improved performance status, and deteriorated QoL was directly associated with worsened performance status for all QoL scales. Results showed that for Global QoL and all functional scales, patients with an Eastern Cooperative Oncology Group (ECOG) performance status score = 1, whereby they were restricted in physically strenuous activity but ambulatory, had worse QoL than patients with an ECOG performance status score = 0, who were fully active and able to carry on all pre-disease performance without restriction. Estimates of ORs characterising QoL changes from baseline to week 6 as predictors of Performance Status changes showed that a 10-point unit change predicted performance status changes for Global QoL (OR:0.81; 95% CI: 0.72–0.90), Role Functioning (OR: 0.83; 95% CI: 0.76–0.90), Emotional Functioning (OR: 0.80; 95% CI: 0.72–0.90). A 5-point unit change predicted performance status changes for Physical Functioning (OR: 0.83; 95% CI: 0.78–0.88). Finally, a 15-point change predicted performance status changes for Cognitive Functioning (OR: 0.81; 95% CI: 0.67–0.98) and Social Functioning (OR:0.82; 95% CI: 0.71–0.94).

Finally, for proximity to death, Bubis et al. [53] found that for individuals with metastatic gastric cancer, odds of reporting elevated scores of anxiety, depression, and general wellbeing were significantly increased in the final 2–4 months of life. In particular, the odds of reporting elevated symptom scores for anxiety, depression and general wellbeing in the last month of life, were 2.11, 2.71 and 3.19 times the odds of reporting elevated scores 6 months before death respectively.

#### Biological factors associated with psychosocial functioning

One study explored biological factors associated with psychosocial functioning in individuals with advanced gastric cancer. Koh et al. [48] investigated the relationship between the brain-derived neurotrophic factor (BDNF) Val66Met polymorphism and coping response to stress in patients diagnosed with advanced gastric cancer. In their study, participants were grouped by genotype into two categories: Val/Val (i.e., Val homozygous group), and Val/Met + Met/Met (i.e., Met allele carrier group). Findings indicate that for individuals with advanced gastric cancer, coping style to cancer significantly differed between the Met allele carrier group and the Val homozygous group, with Met carriers significantly more likely to have a higher Anxious Preoccupation coping style [F = 2.855, df [5, 83], *p* = 0.020] [48]. The correlation coefficients for the Mini-MAC sub-scales for the Met allele carriers’ group and Val homozygote group were 0.23 (anxious preoccupation), 0.07 (Helplessness/Hopelessness), -0.07 (Fatalism), 0.07 (Fighting spirit) and − 0.1703 (Cognitive Avoidance). These were calculated by the review authors using the reports means and SD’s. A significant sex difference was observed between the two groups (*p* = 0.030).

#### Psychological factors associated with psychosocial functioning

One of the six included quantitative studies explored psychological factors associated with psychosocial functioning in individuals with advanced oesophago-gastric cancer. In their study, Rha et al. [49] explored the relationship between self-efficacy (SE) for coping with cancer and Quality of Life amongst individuals with advanced gastric cancer. They found that SE for coping with cancer subscales all demonstrated significant, moderate to strong, positive associations with QoL measured by the FACT-Ga and FACIT-Sp total scores. The exception was the weak association between SE for using spiritual coping and FACT-Ga (0.23). SE for seeking and understanding medical information was the highest ranked subscale, demonstrating moderate associations with emotional (0.55), functional (0.54), and spiritual wellbeing (0.55). Using spiritual coping was the lowest ranked subscale, demonstrating only weak positive associations with social, emotional, and functional well-being, and no association with physical well-being (0.14, p = 0.055) or the gastric cancer subscale (0.14, p = 0.047). However, it did demonstrate a strong association with spiritual well-being (0.71).

#### Macro-level contextual factors associated with psychosocial functioning

Two studies included in this review identified macro level contextual factors which were independently associated with psychosocial functioning in individuals with advanced oesophago-gastric cancer [52, 53]. Bubis et al. [53] explored the relationship between factors (including sex, geographical residence, age, extent of comorbidities, neighbourhood income and year of diagnosis) and moderate-to-severe symptom scores of anxiety, depression and general well-being in metastatic gastric cancer patients. Merchant et al., [52] looked at similar factors (age, sex, SES quintile, cancer site, Charlson Comorbidity Index, distance to nearest chemotherapy hospital, patient location, region) and moderate-to-severe symptom scores of anxiety and depression in both oesophageal and gastric cancer patients. Significant relationships were identified between sex [52, 53] and geographical residence [53]. Bubis et al. [53] reported that females with metastatic gastric cancer had significantly higher odds of reporting anxiety (OR 1.49; 95% CI 1.16–1.93) and depression (OR 1.32; 95% CI 1.00–1.73) when compared with males. No significant difference was found with general well-being between males or females (OR 1.15; 95% CI 0.90–1.46). Similarly, Merchant et al., [52] found that females with advanced oesophageal and gastric cancer were more also likely to report moderate–severe scores in anxiety/depression (OR 1.58, 95% CI 1.21–2.08 [p < 0.01]. In terms of geographical residence, Bubis et al. [53] found that individuals with metastatic gastric cancer living in rural residences had significantly lower odds of reporting depression (OR 0.64; 95% CI 0.44–0.93), compared with individuals living in urban settings, whereas no significant differences were found between geographical residence and anxiety (OR 0.75; 95% CI 0.52–1.08) or overall wellbeing (OR 0.77; 95% CI 0.55–1.08). In contrast Merchant et al., [52] found that for oesophageal and gastric cancer patients, patient location (defined as rural or urban based on postal code at the time of diagnosis) was not found in association with reporting moderate-to-severe symptom scores in anxiety/depression.

### Description of the qualitative evidence

Six qualitative studies provided insight into the challenges that interfere with psychosocial functioning in advanced oesophago-gastric cancer patients. All studies conducted cross-sectional interviews, with three taking a semi-structured approach [19, 50, 51], two taking a narrative approach [18, 46] and one taking an unstructured approach [45]. Findings were pooled using JBI SUMARI with the meta-aggregation approach. 151 findings were extracted with accompanying illustrations. Credibility was assigned to all findings, of which 86 were considered unequivocal, 36 considered credible, and 29 considered unsupported. The unsupported findings were not included in the meta-aggregation. Thus, 122 study findings were included in this review. These findings were aggregated into 15 categories, based on similarity of meaning, and then further aggregated into five synthesised findings: (i) Physical Challenges, with subthemes of weight loss, fatigue and eating difficulties (ii) Psychological Challenges, with subthemes of shock & denial, despair & hopelessness and low mood, (iii) Social Challenges with subthemes of social isolation, loss of independence and close relationships ; (iv) Existential Challenges with subthemes of acceptance and balance and (v) Challenges to Care & Continuity with subthemes of interactions with HCPs, medication adherence and service navigation. The full list of extracted findings with accompanying illustrations and allocated numbering (Supplementary Material 5) and meta-aggregation tables (Supplementary Material 6) are included in the supplementary information.

#### Physical challenges

Four of the six studies described significant physical challenges, as a result of both the illness and treatment, which interfered with the patients’ everyday lives, ultimately negatively influencing their psychosocial functioning [18, 19, 45, 50]. The main physical symptoms reported to impair psychosocial functioning were uncontrollable weightloss [18, 19, 50], eating difficulties [18, 45, 50] and fatigue [19, 50]. For example, patients described unwanted weight loss that they could not control, which altered their identity and sense of self. One patient recalled how their body became so unfamiliar that they lost their sense of who they were, which in turn affected their sense of personal dignity: *“It does something to you, like you are not the same person, like it’s not really you. It’s undignified when you nearly do not know who you are anymore”*(45 p. 434*).* For others, eating difficulties such as *‘difficulty swallowing and pain’*(18 p.298) and ‘*altered taste buds, pain, and vomiting during meals’* (45 p.433) were felt to be emotionally distressing, as patients struggled to come to terms with their altered abilities. For example, one patient alluded to a vicious circle of Feeling hungry but being unable to swallow or keep food down, which further increased their hunger and sense of longing for food, *“I felt like crying. I felt like crying… I could smell it and thought Oh I could try that and knew in myself as soon as I tried it, I couldn’t get it down I would be sick. . .”* (50 p.189). Finally, advanced oesophago cancer patients described fatigue and sleeping problems caused by *‘pain and other physical symptoms such as increased production of mucus coupled with a recommended elevated sleeping posture’* (19 p.5). Ultimately, these challenges caused significant *‘weakness or lack of energy’*(50 p.189) which interfered with their mood and day-to-day lives, as they found themselves *“in a zombie-like state”* [16, p.5].

#### Psychological challenges

Five of the six studies referred to psychological challenges that occurred as a result of an advanced oesophago-gastric cancer diagnosis and its treatment [18, 19, 46, 50, 51], with the diagnosis experienced as a life-altering experience. As individuals learned to navigate their newfound circumstances, challenges to their psychosocial functioning included feelings of shock and denial [18, 50, 51], despair and hopelessness [18, 19, 46, 50] and an inability go find enjoyment in life [19, 46, 50, 51]. In particular, the shock associated with receiving a diagnosis was particularly distressing, with many patients struggling to comprehend their situation and manage the uncertainty of their futures, “*I felt that my life was at an end, why me? Why now?”, “What’s going to happen?”, “Am I going to die?”* (18 p.298). For one patient, feelings of sadness and fear in relation to the uncertainty of the future were experienced in terms of a loss of personal control:

**“***I’m sad and sometimes scared, but it’s probably very human when you do not know what will happen in the future—so, of course, no one can know, but when you have got that disease, then it is all very uncertain…. When I get scared, I’m kind of out of control, and that’s not good, because I want to be in control of myself…, but then I’m completely out of control.”* [46, p.294].

For others, a sense of hopelessness was evident when describing their situation as ‘*waiting for a funeral procession’*(50 p.190) whilst another participant recalled the sense that time was *‘running out”*(46 p.294). Other participants recalled the overwhelming feelings of despair as they attempted to continue with their everyday lives despite the knowledge that their illness would continue to progress until their death, *“I felt like I was slipping into a depression. Even the simplest tasks were overwhelming. I couldn’t handle the future.”* (18 p.298).

#### Social challenges

In five of the six studies, patients with advanced oesophago-gastric cancer reported social limitations as a result of their illness and its side effects [18, 19, 45, 46, 50]. In particular, social isolation and a loss of independence were key consequences of the illness. For several patients, digestive restrictions, a loss of independence and a diminished sense of self-worth, caused them to revaluate their role in society and the home, “*You lose some of your social life if you cannot sit ‘round a table and chat, and I think you lose some of your dignity. It’s nice to enjoy dinner together. But I cannot, so I go home, even though could actually like to stay longer”*(45 p.434*).* Others described feelings of loneliness and social isolation as the Impact of their illness prevented them from socialising in ways they previously would have enjoyed, “*The best thing about Christmas Eve is the roast duck. But I gave it a miss. I didn’t even feel like visiting my son and celebrating Christmas with him. I decided to stay home”* (19 p.4). However, whilst the social experience was primarily negative, for some individuals, their illness enabled them to develop closeness and companionship with family & friends. One patient described how, *“the contact with my family and friends was very important and helped me not to lose faith. I needed to talk about it over and over again.”* (18 p.299).

#### Existential challenges

The existential struggles of individuals with advanced oesophago-gastric cancer were reported in five of the six included studies [18, 19, 46, 50, 51]. For many, this involved participants fluctuating between *‘acceptance of death and not feeling ready, anxiety and ambivalence taking up a large part of everyday life’* (19 p. 6). In particular, patients described feeling conflicted when trying to balance treatment toxicity with their desire to live a fulfilling life, as taking their medication forced them to confront the reality of their situation:

*In short, it is a 10% improvement. It’s a balance with side effects. I wonder whether the side effects are really bad and if the agent is dramatically effective. I will continue to take it at any price while enduring distressing side effects, if it is effective, but it may not be really effective for me, so it’s a balance with side effects* (51 p. 4).

However, for many individuals, *‘accepting their lives as they were’*(51 p. 5) and the desire to *“take one day at a time”* (46 p.294) enabled them to maintain a sense of normality and control over their illness. For one patient, recognising the value of treatment in improving their quality rather quantity of life reduced the existential distress they faced, *“I try to resign myself to the fact that I am going to die, and that now the treatment will make my last days’ worth living.”* (18 p.298).

#### Challenges to care and continuity

The final synthesised finding to arise from the qualitative evidence was that many individuals with advanced oesophago-gastric cancer encountered distressing challenges to accessing support and managing and coordinating their own illness and treatment. This finding evolved from five of the six included studies [18, 19, 45, 50, 51]. In particular, patients described their struggles with medication adherence, often displaying considerable emotional resistance to taking their medication, “I really wish I could skip it. I have such irresistible feelings.” (51 p.5). For others, common challenges included *‘difficulty getting to appointments and navigating the system; health care professionals who do not understand their situation; and lack of support and symptom management’* (19 p.6). However, continuity, structure and communication with HCPs appeared to mitigate some of the distress experienced by patients:

*They [nurses] do almost everything I ask of them, but I do not get the impression they just stand there and say yes and no without listening to what I’m saying. It’s really almost like they are listening to who I am. There’s an atmosphere that makes me feel understood into my very being* (45 p.432*).*

### Synthesis of the quantitative and qualitative findings

Findings from the qualitative and quantitative studies were juxtaposed to explore whether the qualitative findings explored the challenges and adjustment processes of individuals living with advanced oesophago-gastric cancer and whether the quantitative findings supported or contradicted the qualitative findings in their measurement of psychosocial functioning. What emerged was that whilst the qualitative findings provided rich and detailed insight into the challenges that individuals with advanced oesophago-gastric cancer, very few of the qualitative findings were assessed as predictors of psychosocial functioning in the quantitative findings. Several observations are discussed below.

First, patients suggested that oesophago-gastric cancer is characterised by severe symptom burden, which negatively interferes with their psychosocial health. In particular, challenges relating to digestive restrictions, weightloss and fatigue were significant sources of psychosocial suffering for patients. Given that eating and drinking are vital component of daily life, holding both social and emotional connotations, losing control of the ability to swallow appeared to affect multiple aspects of the patient’s life. The consequences of these challenges were measured by some of the quantitative studies which found that higher grades of dysphagia and lower levels of performance status led to greater impairment of QoL.

Second, patients reported the psychological challenges faced by advanced oesophago-gastric cancer, describing feelings of shock, hopelessness, and an inability to find enjoyment in life. Very few psychological factors were explored as predictors of impaired psychosocial functioning in the quantitative evidence, however self-efficacy was identified as a potential protective factor.

Third, patients indicated that the social impact of advanced oesophago-gastric cancer was a central issue for this population, thus social functioning is likely a necessary target for palliative care intervention. Whilst no quantitative findings specifically sought to assess social factors, one study by Chau et al. [54] identified that changes in performance status were associated with statistically significant differences in changes to social functioning. This finding aligns in some ways to the qualitative findings, whereby patients recalled how the loss of independence associated with their illness diminished their self-worth and caused them to revaluate their role in society.

Fourth, patients identified that fostering a state of acceptance and finding balance between treatment toxicity and their desire to live a fulfilling life were important aspects of their adjustment to the illness. These aspects were not measured in the quantitative evidence.

Finally, patients identified several challenges to accessing support and managing and coordinating the illness and treatment. However, quality of communications with HCPs and continuity when navigating the healthcare system appeared to buffer many of these challenges. With respect to satisfaction with care, no quantitative evidence could confirm these findings.

## Discussion

This mixed method systematic review is the first to investigate the psychosocial functioning of individuals with advanced oesophago-gastric cancer and their carers. Despite growing importance in recognising the role of palliative care in promoting better psychosocial outcomes during a patient’s cancer journey [58], this review found only twelve studies that examined psychosocial functioning in individuals with advanced oesophago-gastric cancer, with no studies exploring the carer experience. Research into the psychosocial impact of oesophago-gastric cancer has primarily been explored for survivors and those undergoing curative treatment [8–10] but this review provides new insights into the challenges faced by those with advanced disease. Moreover, despite the small number of included studies, the majority of studies shared commonalities in highlighting the diverse and complex burden of symptoms amongst patients and the subsequent negative impairment of psychosocial functioning in individuals with advanced oesophago-gastric cancer.

Elevated levels of physical symptom burden as a primary source of impaired psychosocial functioning were acknowledged in four of the six qualitative studies and three of the six quantitative studies. Eating difficulties, such as dysphagia and pain when swallowing in particular, were outlined as a key challenge for advanced oesophago-gastric cancer patients, in both the qualitative and quantitative findings. These findings align to a recent systematic review which identified that there are emotional and social consequences associated with advanced cancer patient’s inability to maintain food intake [59] and are consistent with wider literature on the need for good symptom management in oncology to improve quality of life at end of life [60]. Whilst NICE guidelines for the assessment and management of individuals with oesophago-gastric cancer [7] acknowledge the importance of managing digestive-related symptoms in this population, recognition regarding the need for psychosocial support for these issues is missing from recommendations. Consequently, future research and clinical guidelines should pay tribute to the fact that the physical experience of illness is inseparable from the psychosocial functioning of patients in this population. In particular, further consideration should be given to how timely psychological intervention may be needed to reduce digestive related symptoms to maintain or improve the level of psychosocial functioning in individuals with advanced oesophago-gastric cancer. Moreover, the findings provide preliminary rationale for the need for integrated care in this clinical population, whereby the physical experience of illness is viewed as inseparable from its influence on psychosocial and existential functioning [61].

Further to the physical burden experienced by individuals with advanced oesophago-gastric cancer, distressing psychological challenges were identified via all six of the included qualitative studies, with patients describing feelings of shock, hopelessness, and an inability to find enjoyment in life. Aligned to existing research whereby significant and sustained levels of psychological morbidity are experienced in patients with curative oesophago-gastric cancer [8, 11], this review suggests that impaired psychosocial functioning is also common in those with advanced disease. Moreover, proximity to death was identified as a factor associated with worsened psychosocial functioning [53] aligning to evidence that palliative care needs increase and quality of life deteriorates as cancer progresses [17]. Given that promoting quality of life (QOL) at end of life is a major component of palliative and end of life care, this is an important finding for clinical consideration. However, as all of the qualitative studies included in this review were of cross-sectional design, they provide limited insight into how psychosocial functioning worsens over time. Longitudinal qualitative methods would therefore be beneficial to further confirm these quantitative findings and provide greater insight into the trajectory of QOL for this clinical population.

Whilst very few psychological factors were quantitatively explored as predictors of psychosocial functioning, self-efficacy (SE) for coping with cancer was the only predictive psychological factor identified in this review. In wider populations, a meta-analysis found self‐efficacy to be associated with psychological resilience in physically ill individuals [62], however, other studies have suggested that self-efficacy is more likely to be beneficial if the disease is controllable and moderately severe [63]. In addition, recommendations for self-efficacy enhancement to be considered as a key component of psycho-behavioural programs designed to support patients with cancer throughout chemotherapy have been made [64] however little is known about how these programs would benefit individuals receiving treatment with palliative intent. Whilst this information may be useful when considering interventions to improve SE for coping with cancer in advanced oesophago-gastric cancer patients, further evidence is needed to better understand the role of SE for coping with cancer in this clinical population.

Another major finding from this review related to the social consequences of advanced oesophago-gastric cancer, with the illness likely to negatively impair patients’ ability to effectively engage in social interactions. This impairment was particularly linked to patients’ digestive restrictions and loss of independence. Previous studies have shown that physical symptoms can limit opportunities to engage with others, thus declining physical function is often paralleled by increasing social restriction [61]. This aligns to the numerous studies which have shown that advanced cancer can have a substantial negative impact on social engagement, social identity, and social networks [65]. Whilst no quantitative findings specifically sought to assess social factors, one study by Chau et al [54] identified that changes in performance status were associated with statistically significant differences in changes to social functioning. Whilst a loss of independence was underpinned in the qualitative studies by the fear of being viewed as incapable or a burden on their families, individuals also recognised the value of close family support, expressing gratitude for the help and support they received from their loved ones. A similar paradox was recognised in a recent study, where the social world was described as having the potential to both contribute to, or alleviate suffering [66]. Given the significant evidence that social isolation is known to be associated with reduced well-being and increased depression [67], further examination of how social isolation could be reduced for this clinical population is an important avenue for further research. To date, the feasibility of social support interventions for individuals with advanced oesophago-gastric cancer has not been explored, however, interventions that incorporate social support, such as palliative day care and group therapies, have shown potential in improving quality of life in palliative care populations [68]. As such, further research is needed to determine whether similar interventions may benefit those with advanced oesophago-gastric cancer. In addition, whilst the valuable role that friends and family play in helping patients with advanced oesophago-gastric cancer to manage their illness was highlighted in this review, to date, the voice of the carer remains unheard. Future research should therefore seek to gain the informal carer’s perspective, whilst paying attention to their support needs and experiences over time.

The qualitative findings also identified that for individuals with advanced oesophago-gastric cancer, the threat of an advanced diagnosis can prompt an existential struggle, as patients reflect upon their own mortality and the meaning and purpose of life. For many individuals, the ability to accept their circumstances and live flexibly with the changes that were occurring in their life, mitigated many of these struggles by enabling them to sustain what was important to them and maintain a sense of normality. This concept of acceptance as a source of positive adjustment was seen in five of the qualitative studies. This is in line with mounting evidence which suggests that positive psychological states are both attainable and important for individuals living with advanced disease [69–77]. In recent years, interventions which incorporate Acceptance and Commitment Therapy (ACT) have been evaluated in a number of palliative care populations [78]. ACT interventions aim to encourage individuals to accept their circumstances, tolerate their problems and direct behaviours towards living in the present rather than focussing on their fears for the future [79]. A number of pilot studies have demonstrated that ACT can mediate better physical and psychosocial outcomes in individuals with advanced cancer [80–82]. Based on the findings of the review, which identified acceptance as a key factor associated with psychosocial adjustment in this patient population, ACT-based interventions may be particularly relevant in advanced oesophago-gastric cancer populations. As such, work should be done to further understand the relationship between the process of acceptance and psychosocial outcomes in advanced oesophago-gastric cancer patients, to determine the feasibility of delivering ACT-based interventions to this clinical population.

Whilst no quantitative studies sought to assess existential wellbeing, one study did find that using spiritual coping demonstrated a strong association with spiritual well-being [49]. Existential meaning and spirituality are often linked, with spiritual well-being encapsulating both religious well-being and existential well-being [83]. It is widely acknowledged that unmet spiritual and existential needs can lead to heightened physical and emotional suffering at end of life [84]. Consequently, palliative and end of life care guidelines have highlighted the need for greater focus on spiritual and existential support in end of life care [85]. Existing studies have found that therapeutic interventions such as meaning-making group therapy can enable people to better cope with the existential challenges presented by life-threatening illness [86] however little is known about their role in the care of individuals with advanced oesophago-gastric cancer. Whilst this review provides preliminary insight into the existential challenges faced by individuals with advanced oesophago-gastric cancer and the role of spiritual coping, further evidence is needed to identify the existential needs of this clinical population and establish which methods of support may promote existential wellbeing for individuals with advanced oesophago-gastric cancer.

The most common finding from the qualitative studies related to the value of structure and continuity when navigating the healthcare system, supporting the view that continuity of care (COC) can increase the quality of palliative and end-of-life care [87]. Owing to the complex and multifaceted nature of advanced oesophago-gastric cancer, however, the findings revealed several barriers to accessing support and managing and coordinating the illness and treatment. In particular, the logistics of navigating multi-disciplinary care were often felt to be challenging and frustrating, with fractured communication between departments, an inability to establishing lasting therapeutic relationships and a lack of support for medication adherence appearing to influence the patient’s psychosocial functioning. Similar findings have been identified in patients with complex needs [88] and children with sepsis and multiple organ dysfunction-associated immune dysregulation [89]Whilst evidence from the qualitative studies reported several unmet healthcare needs in this clinical population, no quantitative findings could confirm these findings. In light of the multitude of healthcare challenges outlined throughout this review, future research should seek to obtain insight into the care provided to individuals with advanced oesophago-gastric cancer, in relation to the reported unmet care needs and satisfaction with the care provided. HCPs involved in the care and support of advanced oesophago-gastric cancer patients are in a unique position to offer insight into the unmet needs of this patient population, whilst also recognising system related barriers and limitations to healthcare service provision [90]. However, no studies from their perspective were identified for inclusion in this review. As such, future research should seek to gain their perspectives, which in turn should help guide the development of clinical recommendations and improve service accessibility.

A number of factors were identified within the quantitative evidence that were not explored in the qualitative studies. For individuals with advanced oesophago-gastric cancer, macro-level factors, including gender and geographical residence were found to potentially interfere with patients experiences of anxiety, depression and general wellbeing [52, 53]. These findings may aid HCPs in anticipating difficulties in psychosocial functioning in female patients and in those living in urban areas, which can be used to trigger appropriate referral to psycho-oncology services.

Finally, one biological factor was also identified as having a possible association with psychosocial functioning [48], whereby the Met allele of BDNF Val66Met may be predictive of an anxious coping style in patients with advanced gastric cancer. This finding substantiates existing studies which have found that a higher number of Met-alleles are associated with a higher susceptibility for depression and anxiety [91, 92]. However, as this finding was derived from a single study, it should be considered with caution and further exploration is required to understand the link between biological processes and psychosocial functioning in advanced oesophago-gastric cancer patients.

### Strengths and limitations

The use of a convergent segregated mixed-method design enabled the synthesis of the best available evidence in relation to the psychosocial functioning of individuals with advanced oesophago-gastric cancer and their carers. In following the robust JBI methodology throughout the review, the rigor of the study is evident. From the findings, considerable comparisons could be made when comparing the diverse and complex challenges identified from both the qualitative and quantitative components of the review, to established constructs from validated quantitative scales that measure quality of life in this clinical population such as the EORTC QLQ-OG25 [93] therefore reflecting the validity and reliability of the findings in this review. However, several study limitations should also be discussed. Firstly, the main weakness of this study was the paucity of available evidence to address the research objectives. Whilst the qualitative synthesis of this review provides rich insight into the challenges faced by advanced oesophago-gastric cancer patients, as is common of qualitative research in healthcare research [94], the quantitative findings in this review were primarily developed from single studies, thus making it difficult to ensure their generalisability and the lack of prospective designs preclude causal associations. In addition, the significant heterogeneity between the quantitative methods, outcomes and characteristics of the studies proved challenging to the integration of the qualitative and quantitative findings.

Secondly, as all studies were cross-sectional in nature, future research should seek to employ longitudinal methods as psychosocial functioning in cancer patients has been shown to vary across the illness trajectory [95, 96].

Finally, the included studies only provided the patient perspective, thus future studies should investigate the perspective and experiences of informal carers and HCPs to ensure the full care pathway has been explored. Overall, it is evident that this is an under-researched clinical population, thus considerably more work will need to be done to determine the most effective model of care for this clinical population.

## Conclusion

This review has identified several key factors associated with psychosocial functioning in individuals with advanced oesophago-gastric cancer. It also sheds light on the complex and challenging process of adjustment that they experience. Preliminary evidence suggests that alongside complex symptom management, greater continuity of care and psychosocial interventions (such as those which facilitate social support and promote meaning-making, acceptance and self-efficacy) could improve the psychosocial functioning of this patient group. However, given the scarcity of research evidence outlined in this review, further high-quality research is needed to understand how best to support patients and their carers going forward.

### Electronic supplementary material

Below is the link to the electronic supplementary material.

Supplementary Material 1

## Data Availability

The datasets used and/or analysed during the current study are available from the corresponding author on reasonable request.

## Declarations

### Changes to the protocol

Following the initial searches, a decision was made to focus only on published literature and therefore grey literature was not included. The decision was made after the protocol was published, which is therefore a deviation from the protocol. However, it was felt that searching both medical and psychological databases and journals would offer the greatest likelihood of returning papers relevant to the research question whilst remaining practicable within the limits available to this PhD. A decision was also made to exclude all intervention studies, regardless of whether prevalence data on factors associated with psychosocial functioning could be derived (e.g., where quality of life was measured at baseline). This decision was made after the protocol was published; however, it was felt that as intervention efficacy was not being explored, this exclusion criteria would enable the authors to best address the research objectives.

## Abbreviations

JBI: Joanna Briggs Institute
QoL: Quality of life
ACT: Acceptance and Commitment Therapy
HCP: Healthcare professional
