# Supplementary material for: Psychosocial functioning in individuals with advanced oesophago-gastric cancer: a mixed methods systematic review
Source: BMC Palliat Care. 2023 Oct 28;22:164. doi: 10.1186/s12904-023-01288-0 (PMC10612179; doi:10.1186/s12904-023-01288-0)
Supplement: Supplementary file 1 — Supplementary Material 1 [file 12904_2023_1288_MOESM1_ESM.docx]

Supplementary Material 1 – PRISMA 2020 Checklist

Supplementary Material 2 – Finalised Search Strategy

Supplementary Material 3 – Characteristics of Included Studies

Supplementary Material 4 – Methodological Quality of Included Studies

Supplementary Material 5 – List of Study Findings with Illustrations

Supplementary Material 6 – Meta-Aggregation Tables

## Supplementary Material 7**: S**ummary of quantitative findings

## **Supplementary Material 1**: **PRISMA 2020 Checklist**

| Section and Topic | Item # | Checklist item | Location where item is reported |
| --- | --- | --- | --- |
| TITLE | | |  |
| Title | 1 | Identify the report as a systematic review. | 1 |
| ABSTRACT | | |  |
| Abstract | 2 | See the PRISMA 2020 for Abstracts checklist. | 2-3 |
| INTRODUCTION | | |  |
| Rationale | 3 | Describe the rationale for the review in the context of existing knowledge. | 4-5 |
| Objectives | 4 | Provide an explicit statement of the objective(s) or question(s) the review addresses. | 6-7 |
| METHODS | | |  |
| Eligibility criteria | 5 | Specify the inclusion and exclusion criteria for the review and how studies were grouped for the syntheses. | 7-9 |
| Information sources | 6 | Specify all databases, registers, websites, organisations, reference lists and other sources searched or consulted to identify studies. Specify the date when each source was last searched or consulted. | 9 |
| Search strategy | 7 | Present the full search strategies for all databases, registers, and websites, including any filters and limits used. | Supplementary Material 2 |
| Selection process | 8 | Specify the methods used to decide whether a study met the inclusion criteria of the review, including how many reviewers screened each record and each report retrieved, whether they worked independently, and if applicable, details of automation tools used in the process. | 9-10 |
| Data collection process | 9 | Specify the methods used to collect data from reports, including how many reviewers collected data from each report, whether they worked independently, any processes for obtaining or confirming data from study investigators, and if applicable, details of automation tools used in the process. | 10 |
| Data items | 10a | List and define all outcomes for which data were sought. Specify whether all results that were compatible with each outcome domain in each study were sought (e.g., for all measures, time points, analyses), and if not, the methods used to decide which results to collect. | 8-10 |
|  | 10b | List and define all other variables for which data were sought (e.g., participant and intervention characteristics, funding sources). Describe any assumptions made about any missing or unclear information. | 8-9 |
| Study risk of bias assessment | 11 | Specify the methods used to assess risk of bias in the included studies, including details of the tool(s) used, how many reviewers assessed each study and whether they worked independently, and if applicable, details of automation tools used in the process. | 9-10 |
| Effect measures | 12 | Specify for each outcome the effect measure(s) (e.g., risk ratio, mean difference) used in the synthesis or presentation of results. | 13 |
| Synthesis methods | 13a | Describe the processes used to decide which studies were eligible for each synthesis (e.g., tabulating the study intervention characteristics and comparing against the planned groups for each synthesis | 10 |
|  | 13b | Describe any methods required to prepare the data for presentation or synthesis, such as handling of missing summary statistics, or data conversions. | 13 |
|  | 13c | Describe any methods used to tabulate or visually display results of individual studies and syntheses. | 13-14 |
|  | 13d | Describe any methods used to synthesize results and provide a rationale for the choice(s). If meta-analysis was performed, describe the model(s), method(s) to identify the presence and extent of statistical heterogeneity, and software package(s) used. | 13 |
|  | 13e | Describe any methods used to explore possible causes of heterogeneity among study results (e.g., subgroup analysis, meta-regression). | N/A |
|  | 13f | Describe any sensitivity analyses conducted to assess robustness of the synthesized results. | N/A |
| Reporting bias assessment | 14 | Describe any methods used to assess risk of bias due to missing results in a synthesis (arising from reporting biases). | N/A |
| Certainty assessment | 15 | Describe any methods used to assess certainty (or confidence) in the body of evidence for an outcome. | N/A |
| RESULTS | | |  |
| Study selection | 16a | Describe the results of the search and selection process, from the number of records identified in the search to the number of studies included in the review, ideally using a flow diagram. | 11 |
|  | 16b | Cite studies that might appear to meet the inclusion criteria, but which were excluded, and explain why they were excluded. | 11 |
| Study characteristics | 17 | Cite each included study and present its characteristics. | Supplementary Material 3 |
| Risk of bias in studies | 18 | Present assessments of risk of bias for each included study. | 13 (and Supplementary Material 4) |
| Results of individual studies | 19 | For all outcomes, present, for each study: (a) summary statistics for each group (where appropriate) and (b) an effect estimate and its precision (e.g. confidence/credible interval), ideally using structured tables or plots. | Supplementary Material 3 |
| Results of syntheses | 20a | For each synthesis, briefly summarise the characteristics and risk of bias among contributing studies. | N/A |
|  | 20b | Present results of all statistical syntheses conducted. If meta-analysis was done, present for each the summary estimate and its precision (e.g. confidence/credible interval) and measures of statistical heterogeneity. If comparing groups, describe the direction of the effect. | N/A |
|  | 20c | Present results of all investigations of possible causes of heterogeneity among study results. | N/A |
|  | 20d | Present results of all sensitivity analyses conducted to assess the robustness of the synthesized results. | N/A |
| Reporting biases | 21 | Present assessments of risk of bias due to missing results (arising from reporting biases) for each synthesis assessed. | N/A |
| Certainty of evidence | 22 | Present assessments of certainty (or confidence) in the body of evidence for each outcome assessed. | N/A |
| DISCUSSION | | |  |
| Discussion | 23a | Provide a general interpretation of the results in the context of other evidence. | 25-31 |
|  | 23b | Discuss any limitations of the evidence included in the review. | 31-32 |
|  | 23c | Discuss any limitations of the review processes used. | 31 |
|  | 23d | Discuss implications of the results for practice, policy, and future research. | 25-31 |
| OTHER INFORMATION | | |  |
| Registration and protocol | 24a | Provide registration information for the review, including register name and registration number, or state that the review was not registered. | 3 |
|  | 24b | Indicate where the review protocol can be accessed, or state that a protocol was not prepared. | 3 |
|  | 24c | Describe and explain any amendments to information provided at registration or in the protocol. | 33 |
| Support | 25 | Describe sources of financial or non-financial support for the review, and the role of the funders or sponsors in the review. | 34 |
| Competing interests | 26 | Declare any competing interests of review authors. | 34 |
| Availability of data, code, and other materials | 27 | Report which of the following are publicly available and where they can be found: template data collection forms; data extracted from included studies; data used for all analyses; analytic code; any other materials used in the review. | 33 |

## **Supplementary Material 2: Search strategy**

### Table A1: Database: Ovid MEDLINE(R) ALL <1946 to July 06, 2022>

Search Strategy:

| **#** | **Query** | **Results from 6 Jul 2022** |
| --- | --- | --- |
| 1 | exp *Esophageal Neoplasms/ | 48,973 |
| 2 | exp *Stomach Neoplasms/ | 91,829 |
| 3 | exp *Gastrointestinal Neoplasms/ | 366,962 |
| 4 | (Oesophago-gastric Cancer or Oesophago-gastric tumo?r or oesophago-gastric neoplasm* or oesophago-gastric carcinoma* or Esophago-gastric tumo* or Esophago-gastric neoplasm* or Esophago-gastric carcinoma* or Gastro-oesophageal junction cancer* or Gastro- oesophageal junction tumo* or Gastro-oesophageal junction neoplasm* or Gastro-oesophageal carcinoma* or Gastro-esophageal junction cancer* or Gastro- esophageal junction tumo* or Gastro-esophageal junction neoplasm* or Gastro-esophageal carcinoma* or Oesophageal cancer* or oesophageal tumo* or Oesophageal neoplasm* or esophageal cancer* or esophageal tumo* or esophageal neoplasm* or oesophageal carcinoma* or gastric cancer* or gastric tumo* or gastric neoplasm* or gastric carcinoma* or stomach cancer* or stomach tumo* or stomach neoplasm* or stomach carcinoma* or upper GI cancer or upper gastrointestinal cancer).ti,ab,kw. | 126,547 |
| 5 | 1 or 2 or 3 or 4 | 403,016 |
| 6 | exp *"Quality of Life"/ | 107,105 |
| 7 | exp *Adaptation, Psychological/ | 64,703 |
| 8 | exp *Stress, Psychological/ | 94,841 |
| 9 | exp *Psychological Distress/ | 4,038 |
| 10 | exp *Depression/ | 84,502 |
| 11 | exp *Anxiety/ | 50,940 |
| 12 | exp *Psychosocial Support Systems/ | 452 |
| 13 | exp *Patient Satisfaction/ | 37,956 |
| 14 | (Adjust* or Adapt* or Accept* or Satisfaction or Happ* or Optimis* or Well-being or Depress* or Anxi* or Mood or Distress* or Social Phobia or Quality of life or QoL or health-related quality of life or HRQOL or Patient Satisfaction or Psychosocial or Psychological Distress or Psychological Stress or Psychosocial Need or Common Mental Disorder or Stress Disorder* or Emotion* or Wellbeing or well-being or Social Support or coping or Patient Experience* or Lived experience*).ti,ab,kw. | 3,302,670 |
| 15 | 6 or 7 or 8 or 9 or 10 or 11 or 12 or 13 or 14 | 3,394,624 |
| 16 | exp *Neoplasm Metastasis/ | 46,983 |
| 17 | exp *Palliative Care/ | 34,585 |
| 18 | exp *Terminal Care/ | 43,293 |
| 19 | exp *Terminally Ill/ | 3,947 |
| 20 | (Palliative* or Hospice or comfort care or terminal care or terminally ill or end-of-life care or end-stage or advanced disease or advanced cancer or advanced illness or Incurable or unresectable or patient-centred care or supportive care).ti,ab,kw. | 250,377 |
| 21 | 16 or 17 or 18 or 19 or 20 | 327,920 |
| 22 | 5 and 15 and 21 | 3,195 |
| 23 | limit 22 to English language | 2,666 |
| 24 | limit 23 to humans | 2,470 |
| 25 | limit 24 to dt=20200406-20220706 | 291 |

### Table A2: Database: APA PsycInfo <1806 to July 06, 2022>

Search Strategy:

| **#** | **Query** | **Results from 6 Jul 2022** |
| --- | --- | --- |
| 1 | exp Gastrointestinal Disorders/ | 9,340 |
| 2 | exp Esophagus/ | 377 |
| 3 | exp Stomach/ | 401 |
| 4 | exp Gastrointestinal System/ | 2,808 |
| 5 | (Oesophago-gastric Cancer or Oesophago-gastric tumo?r or oesophago-gastric neoplasm* or oesophago-gastric carcinoma* or Esophago-gastric tumo* or Esophago-gastric neoplasm* or Esophago-gastric carcinoma* or Gastro-oesophageal junction cancer* or Gastro- oesophageal junction tumo* or Gastro-oesophageal junction neoplasm* or Gastro-oesophageal carcinoma* or Gastro-esophageal junction cancer* or Gastro- esophageal junction tumo* or Gastro-esophageal junction neoplasm* or Gastro-esophageal carcinoma* or Oesophageal cancer* or oesophageal tumo* or Oesophageal neoplasm* or esophageal cancer* or esophageal tumo* or esophageal neoplasm* or oesophageal carcinoma* or gastric cancer* or gastric tumo* or gastric neoplasm* or gastric carcinoma* or stomach cancer* or stomach tumo* or stomach neoplasm* or stomach carcinoma* or upper GI cancer or upper gastrointestinal cancer).mp. [mp=title, abstract, heading word, table of contents, key concepts, original title, tests & measures, mesh word] | 622 |
| 6 | 1 or 2 or 3 or 4 or 5 | 12,592 |
| 7 | exp Palliative Care/ | 15,788 |
| 8 | exp Terminal Cancer/ | 1,515 |
| 9 | exp Terminally Ill Patients/ | 4,915 |
| 10 | exp "Death and Dying"/ | 44,381 |
| 11 | exp Hospice/ | 3,550 |
| 12 | exp Advance Directives/ | 1,877 |
| 13 | (Palliative* or Hospice or comfort care or terminal care or terminally ill or end-of-life care or end-stage or advanced disease or advanced cancer or advanced illness or Incurable or unresectable or patient-centred care or supportive care).mp. [mp=title, abstract, heading word, table of contents, key concepts, original title, tests & measures, mesh word] | 31,653 |
| 14 | 7 or 8 or 9 or 10 or 11 or 12 or 13 | 71,386 |
| 15 | 6 and 14 | 478 |
| 16 | limit 15 to up=20200406-20220706 | 52 |

### Table A3: Database: Embase <1974 to July 06, 2022>

Search Strategy:

| **#** | **Query** | **Results from 6 Jul 2022** |
| --- | --- | --- |
| 1 | exp *esophagus cancer/ | 51,539 |
| 2 | exp *stomach cancer/ | 89,679 |
| 3 | (Oesophago-gastric Cancer or Oesophago-gastric tumo?r or oesophago-gastric neoplasm* or oesophago-gastric carcinoma* or Esophago-gastric tumo* or Esophago-gastric neoplasm* or Esophago-gastric carcinoma* or Gastro-oesophageal junction cancer* or Gastro- oesophageal junction tumo* or Gastro-oesophageal junction neoplasm* or Gastro-oesophageal carcinoma* or Gastro-esophageal junction cancer* or Gastro- esophageal junction tumo* or Gastro-esophageal junction neoplasm* or Gastro-esophageal carcinoma* or Oesophageal cancer* or oesophageal tumo* or Oesophageal neoplasm* or esophageal cancer* or esophageal tumo* or esophageal neoplasm* or oesophageal carcinoma* or gastric cancer* or gastric tumo* or gastric neoplasm* or gastric carcinoma* or stomach cancer* or stomach tumo* or stomach neoplasm* or stomach carcinoma* or upper GI cancer or upper gastrointestinal cancer).ti,ab,kw. | 166,813 |
| 4 | exp *digestive system cancer/ | 610,809 |
| 5 | 1 or 2 or 3 or 4 | 671,822 |
| 6 | 1 or 2 or 3 | 202,060 |
| 7 | exp *"quality of life"/ | 127,662 |
| 8 | exp *mental stress/ | 70,433 |
| 9 | exp *psychological adjustment/ | 2,748 |
| 10 | exp *psychological well-being/ | 5,860 |
| 11 | exp *wellbeing/ | 19,254 |
| 12 | exp *depression/ | 240,884 |
| 13 | exp *emotional stress/ | 4,061 |
| 14 | exp *anxiety/ | 62,204 |
| 15 | exp *psychological well-being/ | 5,860 |
| 16 | exp *psychosocial care/ | 4,336 |
| 17 | exp *psychosocial environment/ | 1,508 |
| 18 | exp *Psychosocial Adjustment to Illness Scale/ | 4 |
| 19 | exp *psychosocial disorder/ | 4,272 |
| 20 | exp *psychosocial development/ | 247 |
| 21 | (Adjust* or Adapt* or Accept* or Satisfaction or Happ* or Optimis* or Well-being or Depress* or Anxi* or Mood or Distress* or Social Phobia or Quality of life or QoL or health-related quality of life or HRQOL or Patient Satisfaction or Psychosocial or Psychological Distress or Psychological Stress or Psychosocial Need or Common Mental Disorder or Stress Disorder* or Emotion* or Wellbeing or well-being or Social Support or coping or Patient Experience* or Lived experience*).ti,ab,kw. | 4,415,657 |
| 22 | 7 or 8 or 9 or 10 or 11 or 12 or 13 or 14 or 15 or 16 or 17 or 18 or 19 or 20 or 21 | 4,488,962 |
| 23 | exp *cancer palliative therapy/ | 6,413 |
| 24 | exp *palliative therapy/ | 45,520 |
| 25 | exp *advanced cancer/ | 57,132 |
| 26 | exp *terminal care/ | 40,405 |
| 27 | exp *terminally ill patient/ | 3,160 |
| 28 | (Palliative* or Hospice or comfort care or terminal care or terminally ill or end-of-life care or end-stage or advanced disease or advanced cancer or advanced illness or Incurable or unresectable or patient-centred care or supportive care).ti,ab,kw. | 394,537 |
| 29 | 23 or 24 or 25 or 26 or 27 or 28 | 466,583 |
| 30 | 6 and 22 and 29 | 2,891 |
| 31 | 5 and 22 and 29 | 9,754 |
| 32 | limit 30 to (human and English language) | 2,345 |
| 33 | limit 32 to dd=20200406-20220706 | 262 |

### Table A4: Database: CINAHL <1961 to July 06, 2022>

Search Strategy:

| **#** | **Query** | **Results** |
| --- | --- | --- |
| S22 | S21 AND EM 202004- | 235 |
| S21 | S6 AND S14 AND S20 | 1,254 |
| S20 | S15 OR S16 OR S17 OR S18 OR S19 | Display |
| S19 | ""Palliative* OR Hospice OR “comfort care” OR “terminal care” OR “terminally ill” OR “end-of-life care” OR “end-stage” OR “end of life” OR “advanced disease” OR “advanced cancer” OR “advanced illness” OR Incurable OR unresectable OR “patient-centred care” OR supportive care"" | Display |
| S18 | (MM "Neoplasm Metastasis+") | Display |
| S17 | (MM "Terminal Care+") | Display |
| S16 | (MM "Terminally Ill Patients+") | Display |
| S15 | (MM "Palliative Care") | Display |
| S14 | S7 OR S8 OR S9 OR S10 OR S11 OR S12 OR S13 | Display |
| S13 | ""Adjust* OR Adapt* OR Accept* OR Satisfaction OR Happ* OR Optimis* OR Depress* OR Anxi* OR Mood OR Distress* OR “Social Phobia” OR “Quality of life” OR QoL OR “health-related quality of life” OR “HRQOL” OR “Patient Satisfaction” OR “Psychosocial” OR “Psychological Distress” OR “Psychological Stress” OR “Psychosocial Need*” OR “Common Mental Disorder” OR “Stress Disorder*” OR Emotion* OR Wellbeing OR “well-being” OR “Social Support” OR coping OR “Patient Experience*” OR “Lived experience*”" | Display |
| S12 | (MM "Support, Psychosocial+") | Display |
| S11 | (MM "Patient Attitudes") | Display |
| S10 | (MM "Life Experiences+") | Display |
| S9 | (MM "Attitude to Death+") | Display |
| S8 | (MM "Health and Life Quality (Iowa NOC)+") | Display |
| S7 | (MM "Quality of Life+") | Display |
| S6 | S1 OR S2 OR S3 OR S4 OR S5 | Display |
| S5 | “Oesophago-gastric Cancer” OR “Oesophago-gastric tumo?r” OR “oesophago-gastric neoplasm*” OR “oesophago-gastric carcinoma*” OR “Esophago-gastric tumo*” OR “Esophago-gastric neoplasm*” OR “Esophago-gastric carcinoma*” OR “Gastro-oesophageal junction cancer*” OR “Gastro- oesophageal junction tumo*” OR “Gastro-oesophageal junction neoplasm*” OR “Gastro-oesophageal carcinoma*” OR “Gastro-esophageal junction cancer*” OR “Gastro- esophageal junction tumo*” OR “Gastro-esophageal junction neoplasm*” OR “Gastro-esophageal carcinoma*” OR “Oesophageal cancer*” OR “oesophageal tumo*” OR “Oesophageal neoplasm*” OR “esophageal cancer*” OR “esophageal tumo*” OR “esophageal neoplasm*” OR “oesophageal carcinoma*” OR “gastric cancer*” OR “gastric tumo*” OR “gastric neoplasm*” OR “gastric carcinoma*” OR “stomach cancer*” OR “stomach tumo*” OR “stomach neoplasm*” OR “stomach carcinoma*” OR “upper GI cancer” or “upper gastrointestinal cancer” | Display |
| S4 | (MM "Intestinal Neoplasms+") | Display |
| S3 | (MM "Gastrointestinal Neoplasms+") | Display |
| S2 | (MM "Stomach Neoplasms") | Display |
| S1 | (MM "Esophageal Neoplasms") | Display |

## **Supplementary Material 3: Characteristics of Included Studies**

### Table B1: Characteristics of Included Analytical Cross-Sectional Studies

| **Study** | **Setting** | **Country** | **Participant Characteristics** | **Outcomes Measure** | **Summary of associations with psychosocial functioning** |
| --- | --- | --- | --- | --- | --- |
| Brunelli et al, (2000) (Brunelli et al., 2000) | Outpatient | Italy | Sample: n=98 patients with malignant dysphagia.  Gender: male, n=73 female, n=25  Age: range, 59-74  Tumour Location: Esophagocardial: n=93; Other: n=5  Tumour stage: not disclosed  Ethnicity: Italian | Quality of Life (QOL) | - **Dysphagia grade:**   The correlation coefficients for the different relationships with dysphagia grade and QoL were 0.34 (physical functioning), 0.3 (role functioning), 0.29 (global QoL), 0.31 (fatigue).  The results showed that higher grades of dysphagia resulted in higher levels of impairment reported by the patients  *NB: These correlation coefficients were calculated by the review authors, based on the reported p-values.* |
| Rha et al, (2022) (Rha et al., 2022) | Outpatient | South Korea | Sample: n=202 advanced gastric cancer patients who were planning on or currently receiving palliative chemotherapy.  Gender: n=135 male, n=67 female.  Age: mean, 57.2 (SD = 10.3)  Tumour stage: 99% stage 4. | Quality of Life (QOL) | - **Self-Efficacy (SE)**   SE for coping with cancer subscales all demonstrated significant, moderate to strong, positive associations with QoL measured by the FACT-Ga and FACIT-Sp total scores. The exception was the weak association between SE for using spiritual coping and FACT-Ga (ρ = 0.23).  SE for maintaining activity and independence demonstrated a strong positive association with functional well-being (ρ = 0.72) but a moderate association with the rest of the well-being subscales, including the GaCS (ρ = 0.52).  SE for seeking and understanding medical information demonstrated moderate associations with emotional (ρ = 0.55), functional (ρ = 0.54), and spiritual wellbeing (ρ = 0.55).  SE for managing stress and distress demonstrated a strong positive association with emotional (ρ = 0.67) and functional well-being (ρ = 0.67).  SE for managing side effects demonstrated strong positive associations with functional (ρ = 0.69) and emotional well-being (ρ = 0.67), whereas it demonstrated only moderate associations with physical well-being (ρ = 0.46) and GaCS (ρ = 0.49).  SE for accepting cancer/maintaining a positive attitude/making decision demonstrated a strong positive association with functional (ρ = 0.75), emotional (ρ = 0.69), and spiritual well-being (ρ = 0.64).  SE for seeking social support demonstrated moderate associations with spiritual (ρ = 0.61), social (ρ = 0.55), functional (ρ = 0.52), and emotional well-being (ρ = 0.49).  SE for using spiritual coping demonstrated a strong association with spiritual well-being (ρ = 0.71), whereas it demonstrated only weak positive associations with social, emotional, and functional well-being.  Notably, SE for spiritual coping was not associated with physical well-being (ρ = 0.14, p = .055) or GaCS (ρ = 0.14, p = .047) |

### Table B2: Characteristics of Included Cohort Studies

| **Study** | **Setting** | **Country** | **Participant characteristics** | **Groups** | **Outcomes measured** | **Summary of associations with psychosocial functioning** |
| --- | --- | --- | --- | --- | --- | --- |
| Bubis et al (2021) (Bubis et al., 2021) | Outpatient | Canada | Sample: n=788 metastatic gastric patients who received PC and reported at least one ESAS score during the last 6 months of life  Sex; male, n=527; female, n= 261  Age: range, ≤ 50 - ≥ 81  Tumour Location: Gastric cancer  Tumour Stage: metastatic  Ethnicity: Not disclosed | No symptom screening [n = 728]  Received symptom screening [n = 788] | Anxiety, Depression & Overall well-being | The following clinical and disease variables were independently associated with moderate-to-severe symptom scores of anxiety, depression and overall well-being:   - **Sex**   Female gastric cancer decedents had significantly higher odds of reporting anxiety (OR 1.49; 95% CI 1.16–1.93) and depression (OR 1.32; 95% CI 1.00–1.73). No significant difference was found with general well-being (OR 1.15; 95% CI 0.90–1.46)   - **Geographical Residence**   Individuals living in rural residences had significantly lower odds of reporting depression (OR 0.64; 95% CI 0.44–0.93), compared with individuals living in urban settings. No significant differences were found between anxiety (OR0.75; 95% CI 0.52–1.08) or overall wellbeing (OR 0.77; 95% CI 0.55–1.08)   - **Proximity to death**   For all symptoms, odds of reporting elevated scores were significantly increased in the final 2–4 months of life. In the last month of life, the odds of reporting elevated symptom scores for anxiety, depression and general wellbeing were 2.11, 2.71 and 3.19 times the odds of reporting elevated scores 6 months before death respectively.   - **Extent of comorbidities, income**, **year of diagnosis** and **age** were not found in association with reporting moderate-to-severe symptoms of anxiety, depression or overall wellbeing |
| (Chau et al., 2019) | Outpatient | Global West n= 642, East and Southeast Asia n=249, Rest of the world n=128 | Sample: n=1019 participants with previously treated gastric or gastroesophageal junction (GEJ) cancer  Gender: male, n=719; female, n=300  Age: median, 61 (24-87)  Tumour Location: gastric, n=792; gastroesophageal junction, n=227  Tumour Stage: Metastatic  Ethnicity: Not disclosed | N/A | Quality of Life (QOL) | - **Baseline QLQ-C30 scores**   A summary of baseline QLQ-C30 scores by PS showed worse QoL for patients with PS ≥1 for all scales except diarrhoea. No identifiable differences in baseline QLQ-C30 scores were seen among patients based on disease measurability   - **QoL changes associated with PS changes**   Changes in PS were associated with statistically significant differences in changes in most QoL scales, including global QoL, all functional scales and some symptoms. For all scales, positive changes in QoL were directly associated with improved PS, and deteriorated QoL was directly associated with worsened PS.  For the Physical Function Scale, a 5-point change from baseline in QLQ-C30 at Week 6 for ECOG PS group would result in a clinically relevant association (OR = 0.85 at least)  For Global QOL, Role Functioning, Emotional Functioning, Fatigue, Pain and Nausea/Vomiting Scales, a 10-point change from baseline in QLQ-C30 at Week 6 for ECOG PS group would result in a clinically relevant association (OR = 0.85 at least)  For Cognitive Functioning, Social Functioning and Constipation Scales, a 15-point change from baseline in QLQ-C30 at Week 6 for ECOG PS group would result in a clinically relevant association (OR = 0.85 at least)   - **QoL changes associated with BOR**   In most QoL scales, including global QoL, all functional scales and some symptoms, BOR was associated with statistically significant differences in QoL changes. Disease stabilisation or response appeared to maintain or improve QoL, whereas disease progression led to QoL deterioration.  For the Global QOL scale, a 10-point change from baseline in QLQ-C30 at week 6 for best overall response group would result in a clinically relevant association (OR = 0.85 at least)  For Physical Functioning, Role Functioning, Emotional Functioning, Fatigue, and Nausea/Vomiting scales, a 15-point change from baseline in QLQ-C30 at week 6 for best overall response group would result in a clinically relevant association (OR = 0.85 at least)  For Social Functioning, Pain and Appetite Loss scales, a 20-point change from baseline in QLQ-C30 at week 6 for best overall response group would result in a clinically relevant association (OR = 0.85 at least)  Examining these associations by disease measurability demonstrated similar patterns of QoL changes. PD was associated with QoL declines in patients with measurable or non-measurable disease. |
| (Koh et al., 2014) | Outpatient | South Korea | Sample: n=91 participants with newly diagnosed with metastatic or recurrent non-resectable gastric cancer  Gender: male, n=60; female, n=31  Age: Val homozygous group: mean, 56.6 years (SD = 10.3); Met allele carrier group: mean, 58.2 (SD= 12.0)  Tumour location: Gastric adenocarcinoma  Tumour stage: Metastatic, n=75, Recurrent, n=16,  Ethnicity: Korean | Val homozygous group: n=28, (30.8%)  Val/Met + Met/ Met group: n=63, (69.2%) | Mental adjustment and coping styles to cancer | - **The BDNF genetic variant**   Significant differences in coping style were observed between the Val homozygous group and the Met allele carriers (*p*=0.020).    The correlation coefficients for the Mini-MAC sub-scales for the Met allele carriers’ group and Val homozygote group were 0.23 (anxious preoccupation), 0.07 (Helplessness/Hopelessness), -0.07 (Fatalism), 0.07 (Fighting spirit) and -0.1703 (Cognitive Avoidance)  The Met allele carriers had significantly higher Mini-MAC Anxious Preoccupation scores than the Val homozygote group [F= 2.855, df (5, 83), *p*=0.020].  A significant sex difference was observed between the two groups (*p*=0.030).  NB: *These correlation coefficients were calculated using* the means and SDs*, based on an Effect Size Calculator (*<https://www.campbellcollaboration.org/escalc/html/EffectSizeCalculator-R3.php>*)* |
| (Merchant et al., 2021) | Outpatient | Canada | Sample: n= 1497 patients with gastroesophageal cancer with one or more ESAS assessments  Sex; male, n=1076; female, n= 421  Age: mean, 62.6 years (SD = 11.2)  Tumour Location: Esophageal (n=400), Gastric (n=1097)  Tumour Stage: incurable  Ethnicity: Not disclosed | One or more ESAS assessments [n = 1497]  No ESAS assessments [n = 403] | Anxiety, Depression | The following clinical and disease variables were independently associated with reporting moderate-to-severe symptom scores of anxiety/depression:   - **Sex**   Females were more likely to report moderate–severe scores in anxiety/depression (OR 1.58, 95% CI 1.21–2.08 [p<0.01], compared with males)  **Age, SES quintile, cancer site, Charlson Comorbidity Index, distance to nearest chemotherapy hospital, patient location** and **geographical region**, were not found in association with reporting moderate-to-severe symptom scores in anxiety/depression. |

### Table B3: Characteristics of Included Qualitative Studies

| **Study** | **Setting** | **Country** | **Participant characteristics and sample size** | | **Phenomena of interest** | **Methods for data collection and analysis** | **Description of main results** |
| --- | --- | --- | --- | --- | --- | --- | --- |
| (Laursen et al., 2019) | Outpatient | Denmark | Sample: 17 participants with inoperable oesophageal cancer of the squamous cell carcinoma type.  Gender: 7 females, 10 males  Tumour Location: Esophageal  Age: range: 54 - 74 years.  Tumour Stage: incurable  Ethnicity: Not disclosed | | To illuminate the ways in which incurable oesophageal cancer  disrupts the patients’ lives and how the patients experience and adapt to life with the disease. | Semi-Structured interviews and Kvale and Brinkmann’s phenomenological approach | The study showed that patients feel alone with the threat to their lives and everyday existence; they feel isolated due to the inhibiting symptoms of their illness, anxiety, worry and daily losses and challenges. The illness appears to control the patients’ everyday lives while the patients are left alone with existential thoughts on the future. The patients expose a lack of continuity during the course of the illness and treatment and experience loss of identity, community and dignity. |
| (Missel & Birkelund, 2011) | Outpatient | Denmark | Sample: 5 participants with incurable oesophageal cancer  Gender: Not disclosed  Age: range: 43 and 76 years  Tumour Stage: incurable  Ethnicity: Not disclosed | | To explore how patients diagnosed with incurable oesophageal cancer experience living with the illness, and to provide insight into and an understanding of the patients’ situation, reality and phenomena in their life world. | Narrative interviews and Ricoeur’s theory of interpretation. | The study found that incurably ill oesophageal cancer patients find themselves in a complex life situation. The patients’ need for closeness and intimacy and their need to repeatedly talk about their situation is a consistent characteristic in the stories. The patients need the love and support of their family and friends, but also that the nursing staff regarded them as a complete person during their treatment, and that the staff are ready to listen. Findings suggest that incurable disease affects the whole patient, not just objective pain or a functional error. There is a complex association between meaning, body and situation. |
| (Missel & Bergenholtz, 2021) | Inpatient (hospital setting) | Denmark | | Sample: 18 patients with incurable esophageal cancer  Gender: 8 females and 10 males  Age: range - 54 to 75 years  Tumour Stage: incurable  Ethnicity: Not disclosed | To explore the meaning of dignity from the perspective of people living with and hospitalized for incurable esophageal cancer. | Unstructured interviews and Gadamer’s hermeneutical philosophy. | The study identified that the meaning of dignity was revealed as reverential response in care relationships and eating as an undignifying activity. A balance of the healthcare system’s framework with the lifeworld of the patient was significant in preserving dignity and gave patients a sense of reverent response. Patients were unable to eat ordinary daily meals, which affected their perception of own body and identity, including interactions with others. The resulting bodily changes and social consequences were of crucial importance to the perceived dignity. |
| (Missel et al., 2022) | Inpatient (hospital setting) | Denmark | | Sample: 18 patients with incurable esophageal cancer receiving palliative care  Gender: 8 females and 10 males  Age: range - 54 to 75 years.  Tumour Stage: incurable  Ethnicity: Not disclosed | To examine the phenomenon of existential anxiety when living with esophageal cancer in the context of receiving general palliative care in a hospital setting. | Narrative interviews and Ricoeur’s theory of interpretation. | The study revealed that patients experienced existential anxiety at the loss of a future and homeliness when receiving palliative care. Their existence was reduced to the present, with a break in temporal continuity. An anxious mood permeated their entire being-in-the-world in an unhomelike way. Despite this, patients initiated a restoration of home and meaning expressed as a soothing sense of nostalgia that served as an atmospheric, safe space allowing them to inhabit the borderline between past, present, and future. |
| (Yagasaki et al., 2015) | Outpatient | Japan | Sample: 14 participants with advanced gastric cancer who managed their cancer with oral anticancer agents  Gender: 3 females and 11 males  Age: range - 43 and 80 years  Tumour Stage: incurable  Ethnicity: Not disclosed | | To explore the experiences of patients with advanced gastric cancer receiving oral anticancer agents and their perceptions regarding taking their medication. | Semi-structured interviews and Grounded theory, using the constant comparative method | The study showed that patients with advanced gastric cancer experienced inner conflict, in which considerable emotional resistance to taking their medication affected their occasional nonadherent behaviours. The findings suggest that in patient-centred care, it is imperative that healthcare providers understand patients’ inner conflict and inconsistency between their subjective view and behaviour to support patient adherence. |
| (Watt & Whyte, 2003) | Outpatient | Scotland | Sample: 6 participants with incurable oesophageal cancer experiencing dysphagia  Gender: 5 females and 1 male  Age: range - 63 and 85 years  Tumour Stage: incurable  Ethnicity: Not disclosed | | To explore the experience of dysphagia in oesophageal cancer and how it impacts on quality of life. | Semi-structured interviews and Colaizzi’s thematic analysis. | The study revealed that dysphagia is a distressing symptom which has consequences for the patient’s physical, emotional, and social well-being. Participants described feelings of shock or  disbelief when given the diagnosis of cancer. They experienced severe restrictions in their diet because of dysphagia and talked about the upsetting emotional feelings which had arisen as a consequence of dysphagia. Dysphagia was also found to limit the social aspects of the patients’ lives. |

## **Supplementary Material 4: Methodological Quality of Included Studies**

### **Table 1** *Methodological Assessment of included Analytical Cross-Sectional Studies*

| Citation | Q1 | Q2 | Q3 | Q4 | Q5 | Q6 | Q7 | Q8 | % |
| --- | --- | --- | --- | --- | --- | --- | --- | --- | --- |
| Brunelli et al. (2000) | N | Y | Y | Y | U | N | U | Y | 62.5% |
| Rha et al. (2022) | Y | Y | Y | Y | Y | Y | Y | Y | 100% |

*N, no; U, unclear; Y, yes. JBI Critical Appraisal Checklist for analytical cross-sectional studies. Q1. Were the criteria for inclusion in the sample clearly defined? Q2. Were the study subjects and the setting described in detail? Q3. Was the exposure measured in a valid and reliable way? Q4. Were objective, standard criteria used for measurement of the condition? Q5. Were confounding factors identified? Q6. Were strategies to deal with confounding factors stated? Q7. Were the outcomes measured in a valid and reliable way? Q8. Was appropriate statistical analysis used?*

### **Table 2** *Methodological Assessment of included Cohort Studies*

| **Citation** | **Q1** | **Q2** | **Q3** | **Q4** | **Q5** | **Q6** | **Q7** | **Q8** | **Q9** | **Q10** | **Q11** |  |
| --- | --- | --- | --- | --- | --- | --- | --- | --- | --- | --- | --- | --- |
| **Chau *et al.* (2019)** | Y | Y | Y | Y | Y | U | Y | Y | Y | U | Y | 90% |
| **Koh *et al*. (2014)** | Y | Y | Y | Y | Y | U | Y | Y | Y | U | Y | 90% |
| **Bubis *et al*. (2021)** | N | Y | Y | Y | Y | N/A | Y | Y | Y | N/A | Y | 90% |
| **Merchant *et al.* (2021)** | Y | Y | Y | Y | Y | N/A | Y | Y | Y | N/A | Y | 100% |

*N, no; U, unclear; Y, yes. JBI Critical Appraisal Checklist for analytical cohort studies. Q1. Were the two groups similar and recruited from the same population? Q2. Were the exposures measured similarly to assign people to both exposed and unexposed groups? Q3. Was the exposure measured in a valid and reliable way? Q4. Were confounding factors identified? Q5. Were strategies to deal with confounding factors stated? Q6. Were the groups/participants free of the outcome at the start of the study (or at the moment of exposure)? Q7. Were the outcomes measured in a valid and reliable way? Q8. Was the follow up time reported and sufficient to be long enough for outcomes to occur? Q9. Was follow up complete, and if not, were the reasons to loss to follow up described and explored? Q10. Were strategies to address incomplete follow up utilized? Q11. Was appropriate statistical analysis used?*

### **Table 3** *Methodological Assessment of included Qualitative Studies*

| Citation | Q1 | Q2 | Q3 | Q4 | Q5 | Q6 | Q7 | Q8 | Q9 | Q10 |  |
| --- | --- | --- | --- | --- | --- | --- | --- | --- | --- | --- | --- |
| Laursen et al. (2019) | Y | Y | Y | Y | Y | Y | U | Y | Y | Y | 95% |
| Missel & Birkelund (2011) | Y | Y | Y | Y | Y | N | U | Y | Y | Y | 85% |
| Yagasaki et al. (2015) | U | Y | Y | Y | Y | N | U | Y | Y | Y | 85% |
| Watt & Whyte (2003) | U | Y | Y | Y | Y | N | Y | Y | Y | Y | 85% |
| Missel & Bergenholtz, (2021) | Y | Y | Y | Y | Y | Y | U | Y | Y | Y | 95% |
| Missel et al. (2022) | Y | Y | Y | Y | Y | Y | U | Y | Y | Y | 95% |

*N, no; U, unclear; Y, yes. JBI QARI appraisal instruments: critical appraisal checklist for interpretive and critical research: Q 1: Is there Congruity between the stated philosophical perspective and the research methodology? Q 2: Is there congruity between the research methodology and the research question or objectives? Q 3: Is there congruity between the research methodology and the methods used to collect data? Q 4: Is there congruity between the research methodology and the representation and analysis of data? Q 5: Is there congruity between the research methodology and the interpretation of results? Q 6: Is there a statement locating the researcher culturally or theoretically? Q 7: Is the influence of the researcher on the research, and vice-versa, addressed? Q 8: Are participants, and their voices, adequately represented? Q 9: Is the research ethical according to current criteria or, for recent studies, and is there evidence of ethical approval by an appropriate body? Q 10: Do the conclusions drawn in the research report flow from the analysis, or interpretation, of the data*

## **Supplementary Material 5: List of Study Findings with Illustrations**

| **Study (A): Laursen (2019) - 34 findings** | |
| --- | --- |
| Finding (1) | The illness affects the patients’ social interaction, especially social interaction around mealtimes. (U) |
| Illustration | “The best thing about Christmas Eve is the roast duck. But I gave it a miss. I didn’t even feel like visiting my son and celebrating Christmas with him. I decided to stay home.” (p.4) |
| Finding (2) | Some patients feel embarrassed and undignified when eating in the company of others, and they often end up withdrawing from social situations (U) |
| Illustration | “Sometimes I can’t eat food at all, and then I have to throw the food up again, and it’s not nice - and not at all when you are with others - it takes some of your dignity, I think.” (p.4) |
| Finding (3) | As their illness progresses, the patients increasingly isolate themselves, some patients, even more, when fed through a tube. The feeding tube becomes a symbol of their illness. (C) |
| Illustration | One patient report that her grandchildren did not want to sit next to her after she had been given a feeding tube. (p.4) |
| Finding (4) | Others avoid social situations altogether (C) |
| Illustration | One patient commenting that he specifically requests a table in the corner when he is in public places. (p.4) |
| Finding (5) | The patients do not feel up to taking part in family occasions like they used to. (N) |
| Illustration | No illustration |
| Finding (6) | Nor do they feel up to taking an interest in their children or grandchildren’s lives. (N) |
| Illustration | No illustration |
| Finding (7) | It can be hard to share these thoughts, emotions, and worries (U) |
| Illustration | “I don’t share my thoughts with my husband. We’re worn out. He can’t take any more. I don’t really have anyone to talk to.” (p.4) |
| Finding (8) | Others report not feeling able to share their thoughts with either relatives or healthcare professionals. (N) |
| Illustration | No illustration |
| Finding (9) | Some patients cannot cope with their relatives being emotional or expressing their worry or concern and so choose not to involve their social network more than is necessary. (N) |
| Illustration | No illustration |
| Finding (10) | Some patients, however, say that it has done them good to share their thoughts with the medical staff (N) |
| Illustration | No illustration |
| Finding (11) | The patients’ physical symptoms are interfering and controlling factors in their daily lives. The patients talk of how pain relief treatment makes them tired and dulls their senses (C) |
| Illustration | They feel like they are “in a zombie-like state” (p.5) |
| Finding (12) | Because of these side effects the patients sometimes turn down pain relief medication, which affects their mood and enjoyment of life (U) |
| Illustration | “My daughters have told me I’ve become a cross old woman. It’s because I’m in pain. I’m not putting on an act – I look cross because it really hurts”. |
| Finding (13) | The patients also feel alone and left at a “table in the corner” when struggling and coping with the pain. (C) |
| Illustration | “table in the corner” (p.5) |
| Finding (14) | Pain and other physical symptoms such as increased production of mucus coupled with a recommended elevated sleeping posture mean discomfort and sleeping problems. The patients start to feel tired and exhausted in their day-to-day lives (U) |
| Illustration | [The patients] describe how they metaphorically experience walking around like “zombies” and how ordinary, everyday chores, such as cleaning, cooking and gardening seem insurmountable (p.5) |
| Finding (15) | They have to ask others for help, which is not always easy (U) |
| Illustration | "My energy has completely disappeared, so it is hard to get started at home, and my wife has to do everything. I have always been working and it was me doing the gardening, but now … Now it is hard to get dressed. I’m very tired, and I can’t do the same as before, and it takes some of my dignity.” (p.5) |
| Finding (16) | Patients perceive being a burden to others resulting in loss of dignity and a change in their own identity (U) |
| Illustration | “Things have moved fast. Things don’t work anymore. I’m in a lot of pain. I’m limp and I’m dull. I don’t know what to do because I can’t do anything. It is my wife who does the shopping and arranges things. I’m just doing what I’m told. I’m going out with the garbage. It does something to the dignity, when I cannot be the one, I usually was.” (p.5) |
| Finding (17) | Owing to difficulties in swallowing, many patients also lose a great deal of weight, which changes their appearance and can be worrying and upsetting (U) |
| Illustration | “I’ve lost 20 kg since … I’m not sure when. I haven’t been weighing myself because I’ve noticed it from my clothes. One morning I looked at myself in the mirror and thought, there’s something wrong. So, I bought a scale and when I weighed myself, I was shocked.” (p.5) |
| Finding (18) | Weight loss affects patients’ identity through impacting on body image and their everyday functioning resulting in loss of control and confidence in their body which adds to the feeling of being in a “zombie-like state”: (U) |
| Illustration | “After I got the stent [in the oesophagus], I started coughing a lot, and it really hurts, and I can’t eat anything. Usually, it starts at night which means that I don’t get any sleep. So, of course I’m tired all the time. I feel like a zombie or whatever, you know slipping into a sleep and then wake up and then fall asleep again. And when you are in pain and when you don’t sleep at night …, then there isn’t much dignity left.” (p.5) |
| Finding (19) | Mealtimes are for the patients a struggle – a struggle for survival. They become irritable and their daily rhythm becomes disrupted to such an extent that it becomes difficult to be an agent in own life. (N) |
| Illustration | No illustration |
| Finding (20) | Patients might feel like a shadow of themselves banished to “a table in the corner” (C) |
| Illustration | “a table in the corner.” (p.5) |
| Finding (21) | To be in a “zombie-like state” carries a symbolic conveyance of patients being hit by activity loss and being homeless in their own body; it describes the interruption of everyday life caused by pain, exhaustion and illness. (C) |
| Illustration | The “zombie-like state” is an aesthetic for a deep despair in the patients. (p.5) |
| Finding (22) | To maintain usual daily routines and living day to day are a way of clinging to life and, therefore, not relinquishing what they know and who they are. (N) |
| Illustration | No illustration |
| Finding (23) | Patients are also reflecting on life and death. They feel life-threatened, and for some this might lead to a state where they are not able to act (U) |
| Illustration | “You have to get on with your life, which can be difficult when you more or less don’t know any more who you are, when you’ll be here and when you suddenly won’t be here anymore. It’s a different mindset from the mindset you had 10 years ago before you were diagnosed”. (p.5) |
| Finding (24) | Many patients talk about not having much time left to live, but at the same time, they describe their treatment trajectory as if it might provide a cure rather than just relief. (N) |
| Illustration | No illustration |
| Finding (25) | As described earlier physical symptoms dominate the patients’ outlook on life and everyday existence, and often they let their symptoms determine the agenda. As such, activities which the patients previously could be engaged in, seems more difficult because of the illness, and there is a disruption between the patients’ definition of himself/ herself with regard to the past, the present and the anticipated future (N) |
| Illustration | No illustration |
| Finding (26) | The structures in everyday life become disjointed and the planning horizon, which the patients previously have had, shrinks leaving the patients at a “table in the corner” (U) |
| Illustration | “I’ve lost the courage of my life. I’m just sitting and sitting all day, and I’ve never done  that before. And the family …, I don’t see them very often anymore. It really makes me sad. And my daughters have told me that I’ve really changed. The future is pretty uncertain which make it hard to plan anything”. (p.6) |
| Finding (27) | Common to all patients is also a reflection on their own existence and how they should spend the last part of their lives. They oscillate between acceptance of death and not feeling ready, anxiety and ambivalence taking up a large part of everyday life. The patients describe existential fear as a facet of living with the illness (U) |
| Illustration | “I get frightened at night and I daren’t fall asleep. Don’t ask me why because if I think about it rationally it’s like, so you fall asleep and don’t wake up – what’s the big deal. However, fear’s just there.” (p.6) |
| Finding (28) | The patients are questioning if their life has meaning, purpose, or value because there are limits or boundaries on it. Despite the existential questioning, the patients approach their incurable illness in different ways. (N) |
| Illustration | No illustration |
| Finding (29) | Patients want to keep things as normal as possible for as long as they can, taking 1 day at a time and focusing on the present. (U) |
| Illustration | “One day at a time.” (p.6) |
| Finding (30) | Patients in this study experience a lack of continuity during their treatment leaving the patients with a feeling of being left at a “table in the corner.” (C) |
| Illustration | They describe how frustrating it is to meet a new doctor each time and to explain the details of their illness all over again. This lack of continuity and constant repetition of their medical history is wearing, and they explain how burdensome it is to “be a talking medical record”. (p.6) |
| Finding (31) | Patients feel “abandoned and at sea”, unsure of whom to contact if their symptoms increase. It is challenging for them to manage and coordinate their own illness (U) |
| Illustration | “When I started getting worse, I thought, who should I contact? If you rang up, anyone could pick up the phone. That’s what it felt like to me, anyway. It felt like there was no-one specific to refer to, someone who knew my case. I felt abandoned – at sea. There was no-one to talk to.” (p.6) |
| Finding (32) | Some of the issues that were described by the patients when continuity was lacking include: difficulty getting to appointments and navigating the system; health care professionals who do not understand their situation; and lack of support and symptom management. (C) |
| Illustration | Consequently, patients feel “at sea” in the encounter with the health care system. (p.6) |
| Finding (33) | Patients feel that their treatment programme has been put together based on the average patient and fails to take into account their individual situation and needs and the patients are left at a “table in the corner.” (U) |
| Illustration | “I’ve felt totally abandoned or left to myself. The oesophagus is divided into three, when you come to the hospital, you know, three different departments. It makes communication and coordination difficult. Nobody knows my story and I myself have to be the coordinator of everything. I don’t think that it is about caring for ill people, and I’ve felt it’s been unworthy.” (p.6) |
| Finding (34) | Patients found continuity valuable and important, and when they experience continuity in the relationships with health care professionals, they feel having more control over their situation. (U) |
| Illustration | “One person who managed my case in a team with two nurses. And a direct telephone number. It’s been a great help. Now I’d get active if I noticed something myself and thought that something needed to be looked at.” (p.6) |
| **Study B: Missel & Birkelund (2011) – 21 findings** | |
| Finding (1) | The first experience of symptoms for example, difficulty with swallowing and pain. These symptoms indicated to the participant, that something was not as it should be. (U) |
| Illustration | “I didn’t feel much at the beginning, only a little difficulty swallowing and sometimes a burning sensation.” (p.298) |
| Finding (2) | The participants have had the symptoms for some time before they go to the doctor, but only when they are admitted to hospital do, they feel as though they are ill (U) |
| Illustration | “I persuaded myself that it wasn’t serious and just got on with my life.” (p.298) |
| Finding (3) | Denial transforms fear into feelings that are less threatening and easy to overcome (C) |
| Illustration | “Call it denial, but several weeks passed before I realised how serious the situation was.” (p.298) |
| Finding (4) | There is a particular social situation, which incites the participants to seek help. This could for example be that they suddenly realise that a social or personal relation will be disturbed by a symptom, and they seek help, as this distraction has grown too large (U) |
| Illustration | “It all started one evening in May, when I was trying to take a tablet for my hay fever. I nearly choked and couldn’t even drink any water. My husband and I went straight to the A&E.” (p.298) |
| Finding (5) | The participant has begun to suspect something when he contacts the doctor, but it is the final diagnosis that is the actual turning point in the participant’s life. (N) |
| Illustration | No illustration |
| Finding (6) | Several of the participants felt that the doctor at the initial consultation did not take them seriously. This delayed the treatment but also made the participants feel insecure and worried (U) |
| Illustration | “The doctor said that it was gastric acid and that I should just have some tablets. I tried to tell him that it had to be more than that, but he insisted that I try the tablets and see him again if I still had problems. I felt really anxious when I left the doctor.” (p.298) |
| Finding (7) | The diagnosis made the participants aware of the seriousness of the situation causing an existential turning point … Their world falls apart, and the thing they did not think could happen to them has suddenly happened. (U) |
| Illustration | “I felt that my life was at an end, why me? Why now?”, “what’s going to happen?”, “am I going to die?” (p.298) |
| Finding (8) | During this period, participants experienced despair and hopelessness. (U) |
| Illustration | “I felt like I was slipping into a depression. Even the simplest tasks were overwhelming. I couldn’t handle the future.” (p.298) |
| Finding (9) | This period of despair was often followed by periods of hope…. This hope gives the participants the will to get through the difficult treatments ahead. (U) |
| Illustration | “After being in very low spirits for some time, I started to hope that I could survive this terrible disease. It was lovely - I was going home for Christmas and even though I could not eat anything, I was convinced that I still had a good life to look forward to.” (p.298) |
| Finding (10) | When the participants were treated and experienced the accompanying side effects such as nausea, vomiting, fatigue and pain - they became more aware of their body than before. The body suddenly started “communicating” with them in a more direct and threatening way than previously, which is demonstrated in the following statement (U) |
| Illustration | “I could hardly recognise my body after the chemotherapy. It was so different. I was really afraid of myself - my body is decaying, and I can’t do anything about it.” (p.298) |
| Finding (11) | The participants’ disease perception is closely linked to the side effects caused by the treatment they are receiving and what it is doing to their bodies (C) |
| Illustration | “Sometimes I started vomiting before I started the infusion.” (p.298) |
| Finding (12) | After some time, the participants may be told that the disease has spread in spite of their fighting spirit and the strenuous treatment (C) |
| Illustration | “I was told that the cancer had spread.” (p.298) |
| Finding (13) | Participants try to come to terms with their situation and the prospect of dying (U) |
| Illustration | “I have had to make sure that everything is in order, because I know that I am going to die.” (p.298) |
| Finding (14) | Even though they [participants] know that they are close to death, they want to make the most of the life they have left. (U) |
| Illustration | “I try to resign myself to the fact that I am going to die, and that now the treatment will make my last days’ worth living.” (p.298) |
| Finding (15) | Phenomena such as doubt, despair and hopelessness, but also hope, certainty and openness were prominent in this phase of the disease. (U) |
| Illustration | “The cancer was back, and it had spread to my liver (..) I feel that I am trying to take things in my stride, but I must say that both my wife and I were very shocked. We had always kept our hopes up that they could at least keep the cancer at bay, so it was a bit of a let-down for us. I still try to focus on the good years I have had and meet my fate, but it is difficult sometimes.” (p.298) |
| Finding (16) | The ability to appreciate life in spite of serious illness was very typical for the participants. (C) |
| Illustration | Their courage was expressed in an unfailing will to exercise or just keep going by having daily objectives. |
| Finding (17) | Patients greatly appreciated the support and help that they received from family and friends (U) |
| Illustration | “Their support has been unsurpassed.” (p.299) |
| Finding (18) | The stories made it clear just how much the social relations mean to the participants. (U) |
| Illustration | “It meant more to me than the professional care from the nurses and doctors.” (p.299) |
| Finding (19) | The need for togetherness and nearness and to repeatedly talk about their situation was a common feature in their stories. (U) |
| Illustration | “The contact with my family and friends was very important and helped me not to lose faith. I needed to talk about it over and over again.” (p.299) |
| Finding (20) | To struggle through illness without the support of the family was difficult for the participants. The family assisted in various ways, but no matter how it was given, it was of immense importance to the participants. (N) |
| Illustration | No illustration |
| Finding (21) | Despite the support from family and friends, the participants were ready to take on as much responsibility for themselves as possible. Being dependent on others was not described as a positive experience (U) |
| Illustration | "I need help for so many things, and I am not happy about it, as it has always been important for me to be able to take care of myself.” (p.299) |
| **Study C: Watt & Whyte (2003) – 34 findings** | |
| Finding (1) | When given the diagnosis of cancer all of the participants described feelings of shock or disbelief. (U) |
| Illustration | “I was absolutely stunned, I remember lying in my bed, turning my face to the wall after (Consultant) told me and I just cried.” (p.187) |
| Finding (2) | Although dysphagia had been the presenting symptom in all of the participants, no one in the group had anticipated that it could mean anything sinister, like cancer. (C) |
| Illustration | One participant in this study had attributed the problem to a hiatus hernia and had delayed in visiting her own doctor for two months after her swallowing problems had first started. Instead, she had tried taking her husband’s ulcer medication to see if this would help. (p. 187) |
| Finding (3) | Despite not recognizing the significance of dysphagia, all other participants visited their own doctor promptly soon after developing the symptom (N) |
| Illustration | No Illustration |
| Finding (4) | When consulting their doctor two of the participants found that their complaint was not taken seriously. (C) |
| Illustration | The Hospital Consultant believed her to have a hiatus hernia and told her to lose weight. Over several months this participant paid two further visits to her own doctor and to the hospital Consultant. However, her complaint was still not taken seriously, and a correct diagnosis was not made for a further year. (p.187) |
| Finding (5) | The main reason for delay in diagnosis was due to a failing by the health professionals attending two of the participants to recognize the importance of the symptom (N) |
| Illustration | No Illustration |
| Finding (6) | All of the participants had experienced fairly severe restrictions in their diet because of dysphagia and had found it necessary to modify the type of food they ate, which in some instances resulted in choice of food becoming very limited. (U) |
| Illustration | “At first, I was hungry but now I am getting that I don’t care if I eat. It’s not the same at all you don’t enjoy any food, you really don’t enjoy food. The only thing I enjoy is maybe that wee cup-a-soup . . . I maybe have two or three a day if I feel like it you know. Even mashed potatoes done in the lowest way with gravy, I feel them lying.” (p.188) |
| Finding (7) | All of the participants reported that it took them longer than normal to eat meals. (C) |
| Illustration | In some instances, meals were protracted by as much as one hour or in one participant’s case her method of solving the problem was to consistently eat less. (p.188) |
| Finding (8) | The difficulties patients had with swallowing were compounded by other symptoms such as pain on swallowing, and without exception all could relate a bad experience associated with this (U) |
| Illustration | ‘I just took a fork-full, and whether it expanded in me there (pointing to oesophageal area) I don’t know but I was holding onto the work surface and was pressing against my washing machine. I couldn’t even talk. I was drinking water and I was in agony for an hour before all that cleared. That was one terrible night. That was the worst night I ever had; it was then I just watched what I was eating.’ (p.188) |
| Finding (9) | The participants were experiencing problems with painful wind and acid (N) |
| Illustration | No Illustration |
| Finding (10) | All participants said they had choked at some point during their illness and were concerned about this. (C) |
| Illustration | “It takes 3–4 h, that is a fact, before I pull myself together and then I begin to realize there are other things happening which are bad. Very, very, very bad. I am actually frightened to go to bed. I am frightened to go to bed because you feel you are going to die . . . I know I could go to sleep at any point of the day, but I am scared.” (p.188) |
| Finding (11) | Problems with phlegm or mucous (C) |
| Illustration | Problems with phlegm or mucous were described at interview by four of the participants, but were reported with particular severity by two of this number (p.188) |
| Finding (12) | Another symptom of concern was weight loss (C) |
| Illustration | One participant indicated that she was not worried about her weight being too low at present but said at interview she was concerned if her swallowing did not improve, she would lose more weight. (p.189) |
| Finding (13) | Patients described concerns about weakness or lack of energy (C) |
| Illustration | One man said he hadn’t believed it possible to feel so weak, he attributed this to lack of food although in this case his cancer was advanced, and it is impossible to say to what degree his problems with dysphagia contributed to his weakness. (p.189) |
| Finding (14) | The effect of weight loss was not just felt in physical terms, but patients described emotions such as feeling ‘shocked’, or not looking like themselves when they (patients) saw their bodies. (U) |
| Illustration | “Well to be honest it just makes me not want to look at my own body. I hate it . . . “that’s me a blooming skeleton” but no it definitely takes your life away there is no getting away from it.” (p.189) |
| Finding (15) | The upsetting emotional feelings which had arisen as a consequence of dysphagia. The distress caused by the problem is apparent (U) |
| Illustration | “I felt like crying. I felt like crying . . . I could smell it and thought Oh I could try that and knew in myself as soon as I tried it, I couldn’t get it down I would be sick . . . The smell made me that hungry and when I did get it, I couldn’t eat it at all. That was the worst thing about it. I couldn’t eat it and it made me that hungry I was starving I was.” (p.189) |
| Finding (16) | Most of the participants in this study spoke with longing about food they would like to eat but couldn’t (C) |
| Illustration | The researcher noted that she had closed her eyes and was almost savouring the taste of food again. (p.189) |
| Finding (17) | Feelings of fear, insecurity and anxiety about eating (C) |
| Illustration | For some of the participants eating had become a ‘chore’ something they no longer associated with pleasure, one participant even said he found food ‘repulsive’. (p.189) |
| Finding (18) | The patients in this study were aware of the importance of eating and the consequences lack of food could have in terms of both energy and survival (U) |
| Illustration | “I know I need to eat and get nourishment I can feel the energy sapping away. I push myself to do it because I know if I don’t eat, I’ll not be here in a month. I’ll just die of starvation.” (p.189) |
| Finding (19) | Participant’s express feelings that when in hospital their nutritional needs were not met (U) |
| Illustration | “It was just too stodgy and solid, and by the time they got around to giving me some extra gravy or extra stuff I would be going home anyway . . . Well, it was just solid food wasn’t it.” (p.190) |
| Finding (20) | Low mood accompanying dysphagia … Feeling low and downhearted (U) |
| Illustration | ‘I’ll face anything, and I can stand pain, but I am just not the same. I think it is getting on top of me I really do because I am fed up not getting a decent bit of food.’ (p.190) |
| Finding (21) | In some cases, feelings of guilt or hopelessness were evident (U) |
| Illustration | This lady’s sense of hopelessness had been expressed when she described her situation as ‘waiting for a funeral procession’. (p.190) |
| Finding (22) | Positive evaluations of their lives comparing their current health situation more positively than individuals with other health states (U) |
| Illustration | ‘I think I have been quite fortunate, when I think of the things I could have had like Alzheimer’s.” (p.190) |
| Finding (23) | In one instance a lady said that her illness had brought her closer to her family. (N) |
| Illustration | No Illustration |
| Finding (24) | The limitation dysphagia imposed on the social aspects of the patients’ lives (U) |
| Illustration | One participant said she knew she could never go back to a restaurant again, whereas another described the thought of going out for a meal like ‘putting her head in the lion’s den.’ (p.190) |
| Finding (25) | Family life was also affected because of the problem and the patients expressed concerns about this. (C) |
| Illustration | Initially the family of one lady did not know the full extent of her problem and another had actually concealed the problem from her family, only her husband knowing despite the fact that her son also shared the same house. (p.190) |
| Finding (26) | Swallowing difficulties the patients had made them reluctant to eat in front of other people sometimes even close family members and most could relate embarrassing moments caused by the problem and its effect on their lives. (C) |
| Illustration | ‘No, he doesn’t know about the gullet problem because they have their dinner separate. I maybe have mine when I feel like something. I am not at the table with them.’ (p. 190) |
| Finding (27) | In relation to their swallowing problem, patients did express feelings of embarrassment. (N) |
| Illustration | No Illustration |
| Finding (28) | The sense of isolation through the loss of the social role as a result of dysphagia. (U) |
| Illustration | “I don’t see a lot of them [family] which I could do. I could be away on holiday with them, and I don’t visit the same because I am not feeling right. I don’t want to be embarrassed trying to eat . . . I do miss the company.” (p.190/191) |
| Finding (29) | A lack of communication resulted in unnecessary anxiety for the patients (C) |
| Illustration | This was illustrated by two participants who had received letters in the post cancelling their forthcoming therapeutic endoscopies. No explanation had been given and both patients assumed the worst, thinking nothing more could be done for them. (p.191) |
| Finding (30) | Feelings of panic and anxiety (U) |
| Illustration | ‘Oh my God he can do no more, that’s me I’ve had it I’ve had it you know.’ (p. 191) |
| Finding (31) | At the end of this process of clearing the phlegm he felt exhausted. This gentleman was waiting to be admitted to hospital to have more chemotherapy as part of a clinical trial (C) |
| Illustration | "desperate to go in and get something tried" (p.188) |
| Finding (32) | Contrasting experiences were reported as the same participant could also describe positively the support, she had received from the Consultant looking after her (U) |
| Illustration | ‘Mr (Consultant) explains things to you, I feel quite confident when he is there.’ (p.191) |
| Finding (33) | There were concerns about lack of information (C) |
| Illustration | A participant who was anxious about the deterioration in her swallowing felt there had been no opportunity to discuss her future treatment options with anyone. (p.191) |
| **Study D: Yagasaki (2015) – 24 findings** | |
| Finding (1) | The diagnosis of cancer was a shocking event, the participants suddenly realised that life is finite (C) |
| Illustration | "If I do not take medicine, I do not think I will be able to live." (p.3) |
| Finding (2) | To ensure their survival, they had a sense of duty to adhere to medication regimens, because they knew that anticancer agents could potentially extend their lives. Otherwise, their own mortality could become a reality. (U) |
| Illustration | “It is different from common drugs such as cold medicine and stomach medicine. So, I manage to take it because I feel I have to take it. I think I am carefully managing it compare with other drugs.” (p.3) |
| Finding (3) | The participants perceived needs for medication, and used self-motivation to follow their medication regimens (U) |
| Illustration | “I stir myself. It may be my motivation. It’s my obligation. I have to do it. I have to take medicine, so I do.” (p.3) |
| Finding (4) | A trustful relationship with the physician was one of the motivations for continuing treatment (U) |
| Illustration | "I really trust my physician and receive treatment. He is a very good physician for me. I have to do my part too. I cannot betray his trust." (p.4) |
| Finding (5) | Every time the participants took their medication, they had to acknowledge that they were suffering from cancer. It was depressing (U) |
| Illustration | “So far, there are no side effects. But I feel depressed when I take the agent. Maybe it is only a sensory impression” (p.4) |
| Finding (6) | The participants knew that taking medication was not a painful procedure, but they felt uncomfortable doing so, because taking the anticancer agent represented the reality of the situation “I have cancer” (U) |
| Illustration | (The anti-cancer agent is) different from other agents. I am very nervous about taking it. It’s a distinct feeling (p.4) |
| Finding (7) | Participants had considerable emotional resistance to taking their medication (U) |
| Illustration | "Taking TS-1® is no big deal for me, but it is depressing to think that I have to take this anti-cancer agent for four weeks. I tell my friend that it (treatment duration) varies so I have to take it anyway. Take it easy. If it’s like ‘take it or no way,’ I will be depressed" (p.4) |
| Finding (8) | The break between treatments was a relief for the participants, because they did not have to think about cancer. (U) |
| Illustration | I can only forget about the disease during the break. You know, it’s four weeks on and one week off. I say aloud ‘I’m happy!’ (p.4) |
| Finding (9) | The participants provided informed consent to undergo oral chemotherapy, but they still doubted the medication’s efficacy. (U) |
| Illustration | “The efficacy is not so great. It is said to expect about 10% improvement, but I assume that it declines over time.” (p.4) |
| Finding (10) | They suggested that the agent could have been effective for others but not for them. (N) |
| Illustration | No Illustration |
| Finding (11) | Participants were concerned over potential harm from medication…they had strong concerns about side effects and the impact the treatment would have on their lives. (U) |
| Illustration | "In short, it is a 10% improvement. It’s a balance with side effects. I wonder whether the side effects are really bad and if the agent is dramatically effective. I will continue to take it at any price while enduring distressing side effects, if it is effective, but it may not be really effective for me, so it’s a balance with side effects. (p.4) |
| Finding (12) | One woman questioned the significance of oral anticancer agents while balancing the expectation of treatment effects against toxicity (U) |
| Illustration | “So what? For example, to reduce the rate of metastasis by 10%, the agent kills normal cells as well. Is it good for me? I am concerned about it.” (p.4) |
| Finding (13) | As the participants acknowledged the importance of medication, based on their rational beliefs, they strongly denied their attitudes to non-adherence (U) |
| Illustration | “Forget to take medication? Never! It’s different from cold medicine. I don’t think that people who are taking this kind of medicine ever forget it.” (p.4) |
| Finding (14) | The majority of the participants managed the medication in their own ways, and they emphasised how easy taking medication was, stating that they had never broken the rules (U) |
| Illustration | “It’s easy to take medication. I only have to take it after morning and evening meals. I put the medicine on the table. I have never been non-adherent.” (p.4) |
| Finding (15) | Divergence between the head (active participation in therapy) and the heart (temptation to avoid taking medication). The participants were sometimes driven more by emotion than reason (U) |
| Illustration | “I really wish I could skip it (medication). I have such irresistible feelings.” (p.5) |
| Finding (16) | Some patients reported occasionally skipping their medication intentionally (U) |
| Illustration | “I want to have a day off (medication) on Saturdays because I sometimes drink beer in the morning. I try to not to overlap (alcohol and medicine), but sometimes they do overlap, so I skip it (medication).” (p.5) |
| Finding (17) | Other participants also occasionally skipped their medication on purpose, but they justified their nonadherent behaviour by describing it as an exception due to a change in routine or special occasion. (U) |
| Illustration | “I don’t take medication when I entertain my clients or drink alcohol.” (p.5) |
| Finding (18) | The participants also experienced unintentional nonadherence, which mainly involved forgetting to take the medication (U) |
| Illustration | “I usually forget to take medication at night. I sometimes fall asleep right after a meal. I recognise that I’ve forgotten my medication, but it’s too late. I don’t know whether I should take it or not. In such cases, I decide not to take it.” (p.5) |
| Finding (19) | A change in regimen sometimes confused patients. When the regimen was changed, one man continued to take his medication three times, instead of twice, per day (U) |
| Illustration | I thought it was the same, so I took medicine every morning, day, and night. After one week passed, I recognised my mistake, because the medicines were consumed so fast (p.5) |
| Finding (20) | Through the experience of inner conflict, the patients reassessed their lives with cancer, and ultimately accepted their lives as they were. (C) |
| Illustration | "If recurrence does occur, that’s life." (p.5) |
| Finding (21) | Even if the treatment is not effective, they will accept the fact (U) |
| Illustration | “I hope the agent works well, but if it does not work for me, I will accept it.” (p.5) |
| Finding (22) | When the participants acknowledged the situation where there were positive and negative aspects, taking the medication was no longer stressful (C) |
| Illustration | “No more (frustration). Now I feel I am beating it.” (p.5) |
| Finding (23) | The prognosis for her type of cancer was poor, and she struggled to find a solution by balancing the possibility of a 10% improvement against the potential harm to her body. (C) |
| Illustration | She reassessed her values and the meaning of the treatment and, eventually, she found her role in treatment. (p.5) |
| Finding (24) | Once such patients recognise their roles in medication therapy, they will more fully commit to treatment. (C) |
| Illustration | “It’s about my body. So, I have to do what I should do” (p.5) |
| **Study E: Missel and Bergenholtz, (2021) - 23 findings** | |
| Finding (1) | Balancing between the ill person’s lifeworld and the healthcare system’s framework requires flexibility from HCPs. (C) |
| Illustration | *“… the way that they [HCP] are when I need help”* p.432 |
| Finding (2) | Maintaining dignity can, during encounters characterized by a reverent approach, be accommodated by HCPs who manage to be present while keeping their distance and respecting the ill person’s privacy and personal boundaries. (U) |
| Illustration | *“It’s important for my dignity that the staff is good at listening to me and finding out what I need… it’s about being attentive. But, at the same time, they should be*  *professional, so when you are in that kind of situation—a situation where you rely on help—there’s still a certain distance, so they do not get too close or overstep the mark”* p.432 |
| Finding (3) | Participants, though, experience dignity as also involving being listened to. Taking a listening approach offers HCPs the opportunity to find out what the individual needs and then to act accordingly. The approach also allows them to get to know the ill person more deeply, which seems to be highly important to our study participants (U) |
| Illustration | *“They [nurses] do almost everything I ask of them, but I do not get the impression they just stand there and say yes and no without listening to what I’m saying. It’s really almost like they are listening to who I am. There’s an atmosphere that makes me feel understood into my very being” p.432* |
| Finding (4) | Another significant aspect of approaching the participants’ lifeworld in a dignified way is when encounters are not just about disease. When the ill person and HCPs experience a strong rapport, can laugh together, and can have a small talk, it contributes to the ill person’s feelings of worth. (N) |
| Illustration | No illustration |
| Finding (5) | The healthcare system sometimes dominated their encounters with HCPs in ways that significantly affected their sense of dignity. One prominent occurrence that made them not feel valued as an individual was when the treatment trajectory was spread across various hospital departments with multiple contacts, although pervaded by a lack of consistency and continuity. Constantly forced to retell and summarize their treatment and facing frequent disruptions in care, participants felt that nobody really knew them or their illness story. (C) |
| Illustration | *One female participant described how stressful it was that the esophagus was covered by 4 different departments. p.433* |
| Finding (6) | For this participant, the logic of the system appeared to supplant the ill person’s existential situation, impacting the ability to maintain control, consequently affecting the individual’s sense of dignity. (U) |
| Illustration | *“It’s really confusing rushing around to all the departments, totally confusing. I’ve been sent to lots of places; I cannot get my head ‘round it. And when you feel that you are not in control, that takes away some of your dignity” p.433* |
| Finding (7) | Another significant issue affecting participants’ sense of dignity is when HCPs do not have time to listen to them, which leads to a superficial relationship in a non-reverent approach. Participants mentioned how they felt left behind and left to themselves, with no one to count on, and also that they felt they were not being heard or understood. They blamed the healthcare system and not the HCP (U) |
| Illustration | *“All this cost saving, efficiency, and rushing around. No one has time, and that’s not at all dignified. It’s external factors that make them [HCP] behave the way they do. It affects your experience of being a patient when no one has time to listen or to help and, it affects your sense of dignity” p.433* |
| Finding (8) | Waiting time is also a significant aspect of participants’ experience of dignity. When participants have to wait, they find that the system’s framework becomes highly dominant in relation to their own lifeworld. They do not feel taken seriously, they do not feel helped, and they do not feel respected, which can be experienced as feelings of worthlessness. (N) |
| Illustration | No illustration |
| Finding (9) | As a result, the healthcare system may challenge the good intentions of HCPs when they engage in the ill person’s lifeworld, which is why HCPs are required to balance between the system and the lifeworld of patients to treat and care for people  in a dignified way (N) |
| Illustration | No illustration |
| Finding (10) | Dignity is also a matter of whether the information they receive about treatment and care is tailored to the individual in the specific situation. Participants feel worthy when they receive information about what is going on; however, this information should be given using a dialogical, understanding, and reverential approach with HCPs who ask, listen, and answer questions. (C) |
| Illustration | *One participant described how receiving correct information tailored to his situation helped maintain his dignity.* *p.433* |
| Finding (11) | For this participant, the dialogical information approach HCPs took toward him took his thoughts, questions, and lifeworld perspective into account. Feeling heard as an individual influenced his sense of dignity in a vulnerable life situation. (U) |
| Illustration | *“It’s a lot about communication, about whether you as a patient are given proper information and get answers to your questions. You have all sorts of thoughts when you live with the illness. But, as far as I’m concerned, dignity is about whether the information I get is the right information for me, and whether the doctor or nurse gives me answers and understands what I’m asking”* *p.433* |
| Finding (12) | Being met in a way that signalled that the individual patient and his/her issues are important has crucial impact on the perceived dignity. Also, the knowledge, expertise, and carefulness of HCPs are important in the dialogical information for the individual’s sense of dignity. (N) |
| Illustration | No illustration |
| Finding (13) | Information from HCPs can also have a negative impact on the individual’s sense of dignity when misleading or not tailored to the individual, making it incomprehensible. Another aspect of this is when information is given quickly, without empathy. (U) |
| Illustration | *“A doctor met me aside in the corridor and said, ‘We cannot really do anything more for you.’ I was completely shocked. Then her telephone rang, and she was off. It was a busy day, but I could easily have waited a few more days to be told if that had meant things would have been quieter. I do not think that was a dignified way of doing it” p.433* |
| Finding (14) | Eating is essential for survival, and eating is a bodily experience that can occur nearly mindlessly at times; however, taking it for granted was no longer an option for participants, who had to think about what to eat, when to eat, and how to eat every day, all day long (U) |
| Illustration | *“The whole time you are wondering which position is most comfortable. Sometimes I have to sit, sometimes lie. It depends on where the pressure is. At the moment, I have to sit slightly forward on the edge of my chair; otherwise, the stuff comes back up again. It feels undignified not even being able to eat…” p.433* |
| Finding (15) | Other participants described how, unable to even swallow, they were fed through a feeding tube, which significantly affected their sense of dignity, despite its necessity. The desire to participate in food-related activities disappeared, once ordinary daily tasks such as cooking and preparing meals losing all meaning. The feeding tube also hinders ordinary social contact, the threat of loneliness looming in the background (U) |
| Illustration | *“The tube puts me off going to the pub; I mean I’m hardly likely to sit at the bar with this contraption hanging out of my nose. It feels lonely, definitely… But it’s very uncomfortable, undignified even, sitting opposite someone with this thing stuck up your nose.”* *p.433* |
| Finding (16) | Participants described feeling different or having a changed perception of who they were because of eating difficulties. Insufficient intake of food combined with physical changes, such as altered taste buds, pain, eating very slowly, and vomiting during meals, caused weight loss, changing the participants’ physical appearance and performance. Their bodies became unfamiliar, challenging their perception of who they were. (C) |
| Illustration | One participant talked about how difficult it was to explain what it is like to lose 20 kg and have an unrecognizable body *p.433* |
| Finding (17) | The changes to and in the body testify to the disruptive nature of the disease and how the body gradually decays. When the body is changing and languishing, the ill person also changes as a human being and experiences not feeling like the person they used to be, which affects their personal dignity (U) |
| Illustration | *“It does something to you, like you are not the same person, like it’s not really you. It’s undignified when you nearly do not know who you are anymore”* *p.434* |
| Finding (18) | Eating is not just an individual physical activity but one that generally occurs in a social context with others and that is defined by norms and conventions about what and how much to eat and what is acceptable eating behaviour. Participants found that eating in the company of others was a struggle, causing them to feel undignified if they could not get the food down and had to throw up, generally involuntarily. The participants talked about how they always had to be prepared. (U) |
| Illustration | *“Being together with other people when you have big problems eating is really difficult because I have to leave the room. It [the food] gets stuck in your throat and I have to retch. It’s undignified. And I can never tell when it’s going to happen; it’s totally unpredictable. I have to rush to the toilet and be sick… that feels undignified for me”* p.434 |
| Finding (19) | The troubles experienced when eating in the company of others may result in isolation and feelings of loneliness. As a result, eating became the most pronounced, visible sign of the participants’ loss of social activity, seriously impacting their perceived sense of dignity. (C) |
| Illustration | *Participants mentioned how other people excluded them, but also how they kept themselves at a distance from other people in eating situations. p.434* |
| Finding (20) | The feelings of abandonment, loneliness, and worthlessness that can arise when unable to participate in meals in the usual, socially acceptable way. (U) |
| Illustration | “You lose some of your social life if you cannot sit ‘round a table and chat, and I think you lose some of your dignity. It’s nice to enjoy dinner together. But I cannot, so I go home, even though I would actually like to stay longer” *p.434* |
| Finding (21) | Others reassured participants that they were valuable, but they did not always see themselves in this way, or they experienced the opposite and lamented the lack of attention from others, which they assigned to their social network not knowing how to ask or feeling unsure about how to react and show concern, adding to feeling a loss of dignity (N) |
| Illustration | No illustration |
| Finding (22) | Another notable challenge was being unable to eat the meal served, causing feelings of embarrassment and a perceived loss of dignity when family, close friends, or even restaurants had prepared a delicious meal, most of which participants were forced to leave untouched, involuntarily making them appear highly ungrateful. (N) |
| Illustration | No illustration |
| Finding (23) | The inability to eat what is served can be a signifier of illness, reminding both the person who is ill and their social circle about the seriousness of the situation, one in which the frailty of life frequently confronts the participant, negatively affecting their sense of dignity. (N) |
| Illustration | No illustration |
| **Study F: Missel et al (2022) – 16 findings** | |
| Finding (1) | The patients narrate in different ways how they are living their lives in the shadow of the prospect of dying. For them, the process of facing death is filled with anxieties due to certainties as well as uncertainties. (U) |
| Illustration | *“You know, I wonder about what's going to happen…, you wonder with such vehemence…, and it can't help but spread…, so you wonder about whether it'll end up spreading more or…Of course, you worry greatly in a situation like mine. You naturally worry about the future….*” p.294 |
| Finding (2) | Certainties for the participants included the knowledge that their illness would continue to progress until their death, but how this would play out in their lives is expressed as an inherent uncertainty (U) |
| Illustration | *“I have always known that I can die from this.... I know some key figures in the statistics, and it does not look so good…. You know, you go and think about a lot…, your future relationship…, what to take into account and all just suddenly pops up…, there are many thoughts….”* p.294 |
| Finding (3) | For all intents and purposes, the patients' existence is reduced to the present, with a loss of temporality embedded in the question of how long they have left to live (U) |
| Illustration | *“I don't know how much time I have left, so I take one day at a time. That's the only thing I can do…” p.294* |
| Finding (4) | patients were anxious about their very existence (U) |
| Illustration | *You don't know exactly which leg to stand on…. It's a little hard to deal with. I have to take it as it comes and hope for the best, but it's just a tough one to get, I think…I'm down physically and mentally…. I'm scared…it's lying around all the time lurking….* p.294 |
| Finding (5) | For the patients, the experience of loss of a future is not only a distant anticipation but also an immediate fact. This existential loss of a future is, in Heidegger's sense, revealed as being grounded in nothingness. (U) |
| Illustration | *“My daily life has been turned upside down. It's also difficult to explain, but it's like everything, there's obstacles everywhere…. I try to avoid having too many thoughts about how my life will end soon. Because I believe that's the case. I feel like I have aged immensely in the last six months. We all receive a measured amount of time, and perhaps mine is running out….”* p.294 |
| Finding (6) | The mood of the patients in this study shaped their lifeworld perspectives, which means that, when they found themselves in an anxious mood, it coincided with an anxious world (U) |
| Illustration | *“I'm sad and sometimes scared, but it's probably very human when you do not know what will happen in the future—so of course no one can know, but when you have got that disease, then it is all very uncertain…. When I get scared, I'm kind of out of control, and that's not good, because I want to be in control of myself…, but then I'm completely out of control…. “* p.294 |
| Finding (7) | Participant responses to facing their own mortality infiltrated their experience as both provoking anxiety and being objects of anxiety. The anxiety was ever-present, quietly forming an atmosphere of subtle disquiet in the patients' everyday existence. (U) |
| Illustration | *“My life has taken a 180-degree turn, and I simply don't know where I'm at right now…. I've continually tried to keep my spirits up, but in the last month, I haven't felt very well. I don't sleep at night; my mind is whirring*.“ p.294 |
| Finding (8) | This loss of past ways of being-in-the-world reveals the patients' vulnerable nature, and their having to die makes it hard to take part in everyday activities. (N) |
| Illustration | No illustration |
| Finding (9) | Patient narratives about not being at home in their bodies and not being-in-the-world painfully calls for focusing more attention on the issue….For study participants, their bodies appeared as alien beings (being me, yet not me), and this type of obstruction attunes the entire being in-the-world of the ill individuals in an unhomelike way (U) |
| Illustration | *“My family says I have changed, and I probably have.... I have withdrawn. Before I got sick, I took care of my work and was very active. But now…, when I start something, I quickly lose the desire. Every time I try to do something, I cannot.... So, I just sit all day.... I have lost the spark of life... And the grandchildren, I do not see them so often anymore.... I do not have the surplus for it. And the bike…, it does not turn into anything either. So, there has been a drastic change in my everyday life, and I'm scared of it.... I'm just sitting there waiting to die....”* p.295 |
| Finding (10) | The loss of embodiment narrated by this participant, and which can be found in the other patient narratives as well, illuminates how illness makes the patients aware of their bodily nature by creating a disturbance in something, which normally almost completely escapes their attention. The taken-for-granted functions of, for example, breathing and heartbeats become an object of focus for the patients. (U) |
| Illustration | *“I've begun to struggle with anxiety and that puts a damper on me. It comes at night. I don't dare fall asleep. It just sits there, the anxiety that is. My heart begins to beat rapidly, and I have difficulty breathing. I don't really know how to describe what happens. That's why I stay up so late. When I lie down, I wake up after half an hour and get up and walk around. In a way, you don't even know who you are any more, what you are, when you are, and when you might suddenly be gone. All these thoughts have changed compared to what they were like before the illness.”* p.295 |
| Finding (11) | Despite existential loss of a future and facing the world in an anxious mood, the patients narrated how fragments of familiarity existed alongside these unfamiliar dimensions. Although anxiety threatened the sense of being at home, the participants found some strategies for restoring home. (U) |
| Illustration | *“I like to go for walks with the dog in the woods because it helps me mentally to walk peacefully and think about things. Getting a cancer diagnosis is a shock. I've spent so many hours in the woods just walking and thinking, thinking things through. Yes, well, walking and meditating…. “p.295* |
| Finding (12) | Thus, the patients do not have a sense of actual being at home, and as such, their dwelling and thinking do not invoke a genuine homecoming. Against this backdrop, the analysis illuminates how the patients are living in, through, and alongside anxiety while restoring their being at home in such a way that, for brief moments, the world as a whole, in its infinite contingency, is revealed to them. (N) |
| Illustration | No illustration |
| Finding (13) | Illness forces a new form of being-in-the-world on the person when it uncoils the existence usually underpinning the patient's personal life. However, the patients found ways to enter into a dialogue with themselves or others through the restoration of that which were the most personal things upon which they ascribed a sense of homeliness (U) |
| Illustration | *“So, it's my neighbour I share my challenges and thoughts with…he knows a little about how it feels and such, because he himself is also ill. It's nice to have such a friendship so you can get rid of the thoughts, because you cannot just sit and keep it to yourself.” p.295* |
| Finding (14) | Some patients started thinking back in what Heidegger would call a reverie, taking its point of inception in the patients' past (U) |
| Illustration | *“In an instant, my life was turned 180 degrees, and I still do not know exactly where I am now…. I try to keep my mood up, and it helps the most when I do not talk about the disease..., when I talk about other things in my life.... I've had a good life and an exciting life, and it can keep me up when I remember that.” p.295* |
| Finding (15) | Such memories could be understood as nostalgia, as described by Heidegger, who speaks of nostalgic intentionality, which concerns a past of personal experience that matters to us. Through the act of reverie, nostalgia engenders a spatial-temporal continuity that is a central component to a sense of being at home. When the patients in our study talked about themselves and where they were from, nostalgia appeared as an atmospheric *“safe space”* inhabiting a borderline between times, insulating them from a state of peril. (C) |
| Illustration | “safe space” *p.295* |
| Finding (16) | As such, nostalgia has the capacity to enact refamiliarization and might contribute to the possibly soothing work of home restoration. (N) |
| Illustration | No illustration |

## **Supplementary Material 6: Meta-Aggregation Tables**

### Table D1: Meta-synthesis 1 – Physical Functioning

| **Findings (n=21)** | **Categories (n=3)** | **Synthesised Finding** |
| --- | --- | --- |
| (A17) Owing to difficulties in swallowing, many patients also lose a great deal of weight, which changes their appearance and can be worrying and upsetting. [U]  (A18) Weight loss affects patients’ identity through impacting on body image and their everyday functioning resulting in loss of control and confidence in their body which adds to the feeling of being in a “zombie-like state”. [U]  (B10) When the participants were treated and experienced the accompanying side effects such as nausea, vomiting, fatigue and pain - they became more aware of their body than before. The body suddenly started “communicating” with them in a more direct and threatening way than previously. [U]  (C14) The effect of weight loss was not just felt in physical terms, but patients described emotions such as feeling ‘shocked’, or not looking like themselves when they (patients) saw their bodies. [U]  (C12) Another symptom of concern was weight loss. [C]  (B11) The participants’ disease perception is closely linked to the side effects caused by the treatment they are receiving and what it is doing to their bodies. [C] | **Weight Loss –** As a result of advanced oesophago-gastric cancer, individuals found themselves experiencing significant weightloss, which was ‘*worrying and upsetting’* (A17, p.5). | **Synthesised finding 1 - Physical Functioning:**  Individuals with advanced oesophago-gastric cancer face a number of physical challenges that they cannot control. These challenges interfere with their ability to perform instrumental activities of daily living which ultimately has consequences for their physical and psychological health. |
| (B1) The first experience of symptoms for example, difficulty with swallowing and pain. These symptoms indicated to the participant, that something was not as it should be. [U]  (C6) All of the participants had experienced fairly severe restrictions in their diet because of dysphagia and had found it necessary to modify the type of food they ate, which in some instances resulted in choice of food becoming very limited. [U]  (C7) All of the participants reported that it took them longer than normal to eat meals. [C]  (C16) Most of the participants in this study spoke with longing about food they would like to eat but couldn’t. [C]  (C18) The patients in this study were aware of the importance of eating and the consequences lack of food could have in terms of both energy and survival. [U]  (C10) All participants said they had choked at some point during their illness and were concerned about this. [C]  (C8) Problems with phlegm or mucous. [C]  (C17) Feelings of fear, insecurity, and anxiety about eating. [C]  (E14) Eating is essential for survival, and eating is a bodily experience that can occur nearly mindlessly at times; however, taking it for granted was no longer an option for participants, who had to think about what to eat, when to eat, and how to eat every day, all day long (U)  (E16) Participants described feeling different or having a changed perception of who they were because of eating difficulties. Insufficient intake of food combined with physical changes, such as altered taste buds, pain, eating very slowly, and vomiting during meals, caused weight loss, changing the participants’ physical appearance and performance. Their bodies became unfamiliar, challenging their perception of who they were. (C)  (E17) The changes to and in the body testify to the disruptive nature of the disease and how the body gradually decays. When the body is changing and languishing, the ill person also changes as a human being and experiences not feeling like the person they used to be, which affects their personal dignity (U) | **Eating Difficulties** **–** Individuals with advanced oesophago-gastric cancer described challenges such as *‘difficulty swallowing and pain’* when eating. |  |
| (A11) - The patients’ physical symptoms are interfering and controlling factors in their daily lives. The patients talk of how pain relief treatment makes them tired and dulls their senses. [C]  (A14) Pain and other physical symptoms such as increased production of mucus coupled with a recommended elevated sleeping posture mean discomfort and sleeping problems. The patients start to feel tired and exhausted in their day-to-day lives. [U]  (C13) Patients described concerns about weakness or lack of energy. [C]  (C31) At the end of this process of clearing the phlegm he felt exhausted. This gentleman was waiting to be admitted to hospital to have more chemotherapy as part of a clinical trial. [C] | **Fatigue** – Individuals with advanced oesophago cancer described concerns about *‘weakness or lack of energy’* as they attempted to manage their day-to-day lives alongside their illness. |  |

### Table D2: Meta-synthesis 2 - Psychological Functioning

| **Findings (n=24)** | **Categories (n=3)** | **Synthesised Finding** |
| --- | --- | --- |
| A21 - To be in a “zombie-like state” carries a symbolic conveyance of patients being hit by activity loss and being homeless in their own body; it describes the interruption of everyday life caused by pain, exhaustion and illness. [C]  B8 - During this period, participants experienced despair and hopelessness [U]  B9 - This period of despair was often followed by periods of hope…. This hope gives the participants the will to get through the difficult treatments ahead. [U]  B15 - Phenomena such as doubt, despair and hopelessness, but also hope, certainty and openness were prominent in this phase of the disease. [U]  C21 - In some cases, feelings of guilt or hopelessness were evident. [U]  C30 - Feelings of panic and anxiety. [U]  C15 - The upsetting emotional feelings which had arisen as a consequence of dysphagia. The distress caused by the problem is apparent. [U]  F7 - Participant responses to facing their own mortality infiltrated their experience as both provoking anxiety and being objects of anxiety. The anxiety was ever-present, quietly forming an atmosphere of subtle disquiet in the patients' everyday existence. (U)  F10 - The loss of embodiment narrated by this participant, and which can be found in the other patient narratives as well, illuminates how illness makes the patients aware of their bodily nature by creating a disturbance in something, which normally almost completely escapes their attention. The taken-for-granted functions of, for example, breathing and heartbeats become an object of focus for the patients. (U)  F1 - The patients narrate in different ways how they are living their lives in the shadow of the prospect of dying. For them, the process of facing death is filled with anxieties due to certainties as well as uncertainties. (U)  F5 - For the patients, the experience of loss of a future is not only a distant anticipation but also an immediate fact. This existential loss of a future is, in Heidegger's sense, revealed as being grounded in nothingness. (U)  F2 - Certainties for the participants included the knowledge that their illness would continue to progress until their death, but how this would play out in their lives is expressed as an inherent uncertainty (U)  F4 - patients were anxious about their very existence (U) | **Despair and hopelessness** – As their illness progressed, individuals with advanced oesophago-gastric cancer described feelings of despair and hopelessness as they experienced disruption to their daily lives due to *‘pain, exhaustion, and illness’* | **Synthesised finding 2 - Psychological Functioning:**  Being diagnosed with advanced oesophago-gastric cancer is a life-altering experience. As patients learn to navigate their illness, psychological distress is evident as individuals struggle with feelings of shock, hopelessness, and an inability to find enjoyment in life.  . |
| C20 - Low mood accompanying dysphagia … Feeling low and downhearted. [U]  D5 - Every time the participants took their medication, they had to acknowledge that they were suffering from cancer. It was depressing. [U]  A12 - Because of these side effects the patients sometimes turn down pain relief medication, which affects their mood and enjoyment of life. [U]  F6 - The mood of the patients in this study shaped their lifeworld perspectives, which means that, when they found themselves in an anxious mood, it coincided with an anxious world (U) | **Low Mood** - Individuals with advanced oesophago cancer described feeling ‘low and downhearted’ as they navigated the realities of living with a life limiting illness. |  |
| C1 - When given the diagnosis of cancer all of the participants described feelings of shock or disbelief. [U]  D1 - The diagnosis of cancer was a shocking event, the participants suddenly realised that life is finite. [C]  B2 - The participants have had the symptoms for some time before they go to the doctor, but only when they are admitted to hospital do, they feel as though they are ill. [U]  B3 - Denial transforms fear into feelings that are less threatening and easy to overcome. [C]  B7 - The diagnosis made the participants aware of the seriousness of the situation causing an existential turning point … Their world falls apart, and the thing they did not think could happen to them has suddenly happened. [U]  C2 - Although dysphagia had been the presenting symptom in all of the participants, no one in the group had anticipated that it could mean anything sinister, like cancer. [C]  B4 - There is a particular social situation, which incites the participants to seek help. This could for example be that they suddenly realise that a social or personal relation will be disturbed by a symptom and they seek help, as this distraction has grown too large. [U] | **Shock & Denial** – Upon receiving their diagnosis, individuals with advanced oesophago cancer described feelings of shock and disbelief as they were forced to confront the reality of their situation. |  |

### Table D3: Meta-synthesis 3 - Social Functioning

| **Findings (n=24)** | **Categories (n=3)** | **Synthesised Finding** |
| --- | --- | --- |
| A1 - The illness affects the patients’ social interaction, especially social interaction around mealtimes. [U]  A2 - Some patients feel embarrassed and undignified when eating in the company of others, and they often end up withdrawing from social situations. [U]  A3 - As their illness progresses, the patients increasingly isolate themselves, some patients, even more, when fed through a tube. The feeding tube becomes a symbol of their illness. [C]  A4 - Others avoid social situations altogether [C]  A13 - The patients also feel alone and left at a “table in the corner” when struggling and coping with the pain. [C]  A20 - Patients might feel like a shadow of themselves banished to “a table in the corner”  [C]  C28 - The sense of isolation through the loss of the social role as a result of dysphagia. [U]  C26 - Swallowing difficulties the patients had made them reluctant to eat in front of other people sometimes even close family members and most could relate embarrassing moments caused by the problem and its effect on their lives. [C]  C24 - The limitation dysphagia imposed on the social aspects of the patients’ lives. [U]  A26 - The structures in everyday life become disjointed and the planning horizon, which the patients previously have had, shrinks leaving the patients at a “table in the corner” [U]  E18 - Eating is not just an individual physical activity but one that generally occurs in a social context with others and that is defined by norms and conventions about what and how much to eat and what is acceptable eating behaviour. Participants found that eating in the company of others was a struggle, causing them to feel undignified if they could not get the food down and had to throw up, generally involuntarily. The participants talked about how they always had to be prepared. (U)  E19 - The troubles experienced when eating in the company of others may result in isolation and feelings of loneliness. As a result, eating became the most pronounced, visible sign of the participants’ loss of social activity, seriously impacting their perceived sense of dignity. (C)  E20 - The feelings of abandonment, loneliness, and worthlessness that can arise when unable to participate in meals in the usual, socially acceptable way. (U)  F9 - Patient narratives about not being at home in their bodies and not being-in-the-world painfully calls for focusing more attention on the issue….For study participants, their bodies appeared as alien beings (being me, yet not me), and this type of obstruction attunes the entire being in-the-world of the ill individuals in an unhomelike way (U)  (E15) Other participants described how, unable to even swallow, they were fed through a feeding tube, which significantly affected their sense of dignity, despite its necessity. The desire to participate in food-related activities disappeared, once ordinary daily tasks such as cooking and preparing meals losing all meaning. The feeding tube also hinders ordinary social contact, the threat of loneliness looming in the background (U) | **Social Isolation** - Individuals with advanced oesophago-gastric cancer described feelings of loneliness and isolation as the impact of their illness prevented them from socialising in ways they previously would have. | **Synthesised finding 3 - Social Functioning:**  Whilst individuals with advanced oesophago-gastric cancer value closeness and companionship with family & friends, the illness impairs their ability to effectively engage in social interactions. Ultimately this threatens their identity and sense of self, and they struggle with feelings of loneliness and isolation. |
| A15 - They have to ask others for help, which is not always easy. [U]  A16 - Patients perceive being a burden to others resulting in loss of dignity and a change in their own identity. [U]  B21 - Despite the support from family and friends, the participants were ready to take on as much responsibility for themselves as possible. Being dependent on others was not described as a positive experience. [U] | **Loss of independence -** As their illness progressed, individuals with advanced oesophago-gastric cancer found themselves having to rely on others, which diminished their self-worth, and caused them to revaluate their role in society and the home. |  |
| B17 - Patients greatly appreciated the support and help that they received from family and friends. [U]  B18 - The stories made it clear just how much the social relations mean to the participants. [U]  B19 - The need for togetherness and nearness and to repeatedly talk about their situation was a common feature in their stories. [U]  A7 - It can be hard to share these thoughts, emotions, and worries. [U]  C25 - Family life was also affected because of the problem and the patients expressed concerns about this. [C]  F13 - Illness forces a new form of being-in-the-world on the person when it uncoils the existence usually underpinning the patient's personal life. However, the patients found ways to enter into a dialogue with themselves or others through the restoration of that which were the most personal things upon which they ascribed a sense of homeliness (U) | **Close Relationships -** Close relationships with friends and family played an important role throughout the advanced oesophago-gastric cancer trajectory. |  |

### Table D4: Meta-synthesis 4 - Existential Functioning

| **Findings (n=23)** | **Categories (n=3)** | **Synthesised Finding** |
| --- | --- | --- |
| A23 - Patients are also reflecting on life and death. They feel life-threatened, and for some this might lead to a state where they are not able to act. [U]  A27 *-* Common to all patients is also a reflection on their own existence and how they should spend the last part of their lives. They oscillate between acceptance of death and not feeling ready, anxiety and ambivalence taking up a large part of everyday life. The patients describe existential fear as a facet of living with the illness. [U]  B12 - After some time, the participants may be told that the disease has spread in spite of their fighting spirit and the strenuous treatment. [C]  B13 - Participants try to come to terms with their situation and the prospect of dying. [U]  B14 - Even though they [participants] know that they are close to death, they want to make the most of the life they have left. [U]  C22 - Positive evaluations of their lives comparing their current health situation more positively than individuals with other health state. [U]  D20 - Through the experience of inner conflict, the patients reassessed their lives with cancer, and ultimately accepted their lives as they were. [C]  D21 - Even if the treatment is not effective, they will accept the fact. [U]  D24 - Once such patients recognise their roles in medication therapy, they will more fully commit to treatment. [C]  D22 - When the participants acknowledged the situation where there were positive and negative aspects, taking the medication was no longer stressful. [C]  B16 - The ability to appreciate life in spite of serious illness was very typical for the participants. [C]  A29 - Patients want to keep things as normal as possible for as long as they can, taking 1 day at a time and focusing on the present. [U]  F11 - Despite existential loss of a future and facing the world in an anxious mood, the patients narrated how fragments of familiarity existed alongside these unfamiliar dimensions. Although anxiety threatened the sense of being at home, the participants found some strategies for restoring home. (U)  F14 - Some patients started thinking back in what Heidegger would call a reverie, taking its point of inception in the patients' past (U)  F15 - Such memories could be understood as nostalgia, as described by Heidegger, who speaks of nostalgic intentionality, which concerns a past of personal experience that matters to us. Through the act of reverie, nostalgia engenders a spatial-temporal continuity that is a central component to a sense of being at home. When the patients in our study talked about themselves and where they were from, nostalgia appeared as an atmospheric *“safe space”* inhabiting a borderline between times, insulating them from a state of peril. (C)  F3- For all intents and purposes, the patients' existence is reduced to the present, with a loss of temporality embedded in the question of how long they have left to live (U) | **Acceptance** - Once aware of their prognosis, individuals with advanced oesophago-gastric cancer described a process of fostering acceptance and maintaining wellbeing under difficult and severe circumstances. | **Synthesised finding 4 – Existential Functioning:**  For advanced oesophago-gastric cancer patients, the threat of an advanced diagnosis prompts an existential struggle, as individuals confront the uncertainty of their future alongside their endeavours to live in the present and foster acceptance of their circumstances. |
| D6 - The participants knew that taking medication was not a painful procedure, but they felt uncomfortable doing so, because taking the anticancer agent represented the reality of the situation “I have cancer”. [U]  D8 - The break between treatments was a relief for the participants, because they did not have to think about cancer. [U]  D9 - The participants provided informed consent to undergo oral chemotherapy, but they still doubted the medication’s efficacy. [U]  D11 - Participants were concerned over potential harm from medication…they had strong concerns about side effects and the impact the treatment would have on their lives. [U]  D12 - One woman questioned the significance of oral anticancer agents while balancing the expectation of treatment effects against toxicity. [U]  D15 - Divergence between the head (active participation in therapy) and the heart (temptation to avoid taking medication). The participants were sometimes driven more by emotion than reason. [U]  D23 - The prognosis for her type of cancer was poor, and she struggled to find a solution by balancing the possibility of a 10% improvement against the potential harm to her body. [C] | **Balance** - Throughout their journey, individuals with advanced oesophago-gastric cancer described feeling conflicted when trying to balance treatment toxicity with their desire to live a fulfilling life. |  |

### Table D5: Meta-synthesis 5 – Care & Continuity

| **Findings (n=30)** | **Categories (n=3)** | **Synthesised Finding** |
| --- | --- | --- |
| A34 - Patients found continuity valuable and important, and when they experience continuity in the relationships with health care professionals, they feel having more control over their situation. [U]  B6 - Several of the participants felt that the doctor at the initial consultation did not take them seriously. This delayed the treatment but also made the participants feel insecure and worried. [U]  C4 - When consulting their doctor two of the participants found that their complaint was not taken seriously. [C]  C29 - A lack of communication resulted in unnecessary anxiety for the patients. [C]  C32 - Contrasting experiences were reported as the same participant could also describe positively the support, she had received from the Consultant looking after her. [U]  D4 - A trustful relationship with the physician was one of the motivations for continuing treatment. [U]  C33 - There were concerns about lack of information. [C]  E1 - Balancing between the ill person’s lifeworld and the healthcare system’s framework requires flexibility from HCPs. [C]  E2 - Maintaining dignity can, during encounters characterized by a reverent approach, be accommodated by HCPs who manage to be present while keeping their distance and respecting the ill person’s privacy and personal boundaries. (U)  E3 - Participants, though, experience dignity as also involving being listened to. Taking a listening approach offers HCPs the opportunity to find out what the individual needs and then to act accordingly. The approach also allows them to get to know the ill person more deeply, which seems to be highly important to our study participants (U)  E7 - Another significant issue affecting participants’ sense of dignity is when HCPs do not have time to listen to them, which leads to a superficial relationship in a non-reverent approach. Participants mentioned how they felt left behind and left to themselves, with no one to count on, and also that they felt they were not being heard or understood. They blamed the healthcare system and not the HCP (U)  E10 - Dignity is also a matter of whether the information they receive about treatment and care is tailored to the individual in the specific situation. Participants feel worthy when they receive information about what is going on; however, this information should be given using a dialogical, understanding, and reverential approach with HCPs who ask, listen, and answer questions. (C)  E11 - For this participant, the dialogical information approach HCPs took toward him took his thoughts, questions, and lifeworld perspective into account. Feeling heard as an individual influenced his sense of dignity in a vulnerable life situation. (U)  E13 - Information from HCPs can also have a negative impact on the individual’s sense of dignity when misleading or not tailored to the individual, making it incomprehensible. Another aspect of this is when information is given quickly, without empathy. (U) | **Interactions with Healthcare Professionals** –Individuals with advanced oesophago-gastric cancer valued continuity and strong communication in their relationships with individual healthcare professionals, however this was not always experienced. | **Synthesised finding 5 – Care & Continuity:**  Individuals with advanced oesophago-gastric cancer encounter several challenges to accessing support and managing and coordinating their own illness and treatment. They value structure and continuity which enables them to feel in control as they navigate their advanced oesophago-gastric cancer journey. |
| D2 - To ensure their survival, they had a sense of duty to adhere to medication regimens, because they knew that anticancer agents could potentially extend their lives. Otherwise, their own mortality could become a reality. [U]  D14 - The majority of the participants managed the medication in their own ways, and they emphasised how easy taking medication was, stating that they had never broken the rules. [U]  D7 - Participants had considerable emotional resistance to taking their medication. [U]  D16 - Some patients reported occasionally skipping their medication intentionally. [U]  D17 - Other participants also occasionally skipped their medication on purpose, but they justified their nonadherent behaviour by describing it as an exception due to a change in routine or special occasion. [U]  D18 - The participants also experienced unintentional nonadherence, which mainly involved forgetting to take the medication. [U]  D13 - As the participants acknowledged the importance of medication, based on their rational beliefs, they strongly denied their attitudes to non-adherence. [C]  D3 - The participants perceived needs for medication and used self-motivation to follow their medication regimens. [U]  D19 - A change in regimen sometimes confused patients. When the regimen was changed, one man continued to take his medication three times, instead of twice, per day. [U] | **Medication Adherence** - Individuals with advanced oesophago-gastric cancer recognise the importance of adhering to their medication regimes, however some participants described struggling with adherence. |  |
| A30 - Patients in this study experience a lack of continuity during their treatment leaving the patients with a feeling of being left at a “table in the corner.” [C]  A31 - Patients feel “abandoned and at sea”, unsure of whom to contact if their symptoms increase. It is challenging for them to manage and coordinate their own illness. [U]  A33 - Patients feel that their treatment programme has been put together based on the average patient and fails to take into account their individual situation and needs and the patients are left at a “table in the corner.” [U]  A32 - Some of the issues that were described by the patients when continuity was lacking include: difficulty getting to appointments and navigating the system; health care professionals who do not understand their situation; and lack of support and symptom management. [C]  C19 - Participant’s express feelings that when in hospital their nutritional needs were not met. [U]  E5 - The healthcare system sometimes dominated their encounters with HCPs in ways that significantly affected their sense of dignity. One prominent occurrence that made them not feel valued as an individual was when the treatment trajectory was spread across various hospital departments with multiple contacts, although pervaded by a lack of consistency and continuity. Constantly forced to retell and summarize their treatment and facing frequent disruptions in care, participants felt that nobody really knew them or their illness story. (C)  E6 - For this participant, the logic of the system appeared to supplant the ill person’s existential situation, impacting the ability to maintain control, consequently affecting the individual’s sense of dignity. (U) | **Service Navigation -** Individuals with advanced oesophago-gastric cancer described feeling *“abandoned and at sea”* during their encounters with the healthcare system. |  |

## **Supplementary Material 7: S**ummary of quantitative findings

| **Study** | **Summary of associations with psychosocial functioning** |
| --- | --- |
| **Brunelli et al., (2000)** | **Relationship between Dysphagia and QoL**  *Correlation coefficients between dysphagia and quality of life domains:*   - Dysphagia x QoL (physical functioning): r = 0.34 - Dysphagia x QoL (role functioning): r = 0.30 - Dysphagia x QoL (global QoL): r = 0.29 - Dysphagia x QoL (fatigue): r = 0.31   *Note:* These correlation coefficients were calculated by the review authors, based on the reported p-values and are therefore approximations. |
| **Rha et al., (2022)** | **Relationship between Self-efficacy for Coping with Cancer and QoL**  *Correlation coefficients between self-efficacy for coping with cancer and quality of life domains*   \|  \| SE in Maintaining Activity and Independence \| SE in Seeking and Understanding Medical Information \| SE in managing Stress and Distress \| SE in Managing Side Effects \| SE in Accepting Cancer/ Maintaining a Positive Attitude/ Making Decision \| SE in Seeking Support \| SE in Using Spiritual Coping \| \| --- \| --- \| --- \| --- \| --- \| --- \| --- \| --- \| \| Physical wellbeing \| 0.47 \| 0.34 \| 0.39 \| 0.46 \| 0.46 \| 0.33 \| 0.14 \| \| Social wellbeing \| 0.51 \| 0.43 \| 0.50 \| 0.51 \| 0.57 \| 0.55 \| 0.30 \| \| Emotional wellbeing \| 0.56 \| 0.55 \| 0.68 \| 0.67 \| 0.69 \| 0.49 \| 0.29 \| \| Functional wellbeing \| 0.72 \| 0.54 \| 0.67 \| 0.69 \| 0.75 \| 0.52 \| 0.24 \| \| Spiritual wellbeing \| 0.58 \| 0.55 \| 0.59 \| 0.56 \| 0.64 \| 0.61 \| 0.71 \| \| FACT-ga Total \| 0.52 \| 0.30 \| 0.45 \| 0.49 \| 0.50 \| 0.31 \| 0.14 \| \| FACIT-Sp \| 0.66 \| 0.48 \| 0.62 \| 0.67 \| 0.70 \| 0.49 \| 0.23 \| \| GaCS \| 0.73 \| 0.62 \| 0.71 \| 0.73 \| 0.80 \| 0.66 \| 0.49 \|   *Note:*  * FACT-Ga = Functional Assessment of Cancer Therapy-Gastric.  * FACIT-Sp = Functional Assessment of Chronic Illness Therapy – Spiritual Well being  * GaCS = Gastric Cancer Subscale |
| **Bubis et al., (2021)** | **Relationship between patient characteristics and anxiety, depression, overall wellbeing**  *Odds ratio of score > 4 (moderate-to-severe) by ESAS symptom (anxiety, depression, overall wellbeing)*   \| Covariate \| Anxiety \| Depression \| Overall wellbeing \| \| --- \| --- \| --- \| --- \| \|  \| OR (95% CI) \| OR (95% CI) \| OR (95% CI) \| \| Month prior to death  6  5  4  3  2  1 \| 1 (ref)  1.00 (0.79–1.28)  1.04 (0.83–1.31)  1.23 (0.98–1.56)  1.38 (1.07–1.77)  2.11 (1.57–2.83) \| 1 (ref)  1.03 (0.81–1.32)  1.16 (0.90–1.48)  1.24 (0.95–1.61)  1.69 (1.29–2.20)  2.71 (2.00–3.68) \| 1 (ref)  1.08 (0.85–1.38)  1.24 (0.96–1.61)  1.44 (1.10–1.88)  1.69 (1.30–2.19)  3.19 (2.25–4.52) \| \| Sex  Male  Female \| 1 (ref)  1.49 (1.16–1.93) \| 1 (ref)  1.32 (1.00–1.73) \| 1 (ref)  1.15 (0.90–1.46) \| \| Age  18–50  51–60  61–70  71–80  ≥ 81 \| 0.89 (0.61–1.29)  1 (ref)  1.22 (0.86–1.73)  1.00 (0.69–1.43)  0.94 (0.59–1.48) \| 0.79 (0.52–1.19)  1 (ref)  0.99 (0.67–1.41)  0.91 (0.64–1.31)  0.87 (0.54–1.39) \| 0.84 (0.59–1.21)  1 (ref)  0.99 (0.71–1.37)  0.99 (0.71–1.38  0.93 (0.60–1.43 \| \| Comorbidity burden  Low  Moderate  High \| 1 (ref)  0.74 (0.43–1.25)  0.92 (0.53–1.58) \| 1 (ref)  1.12 (0.62–2.01)  1.32 (0.72–2.40) \| 1 (ref)  1.15 (0.69–1.93)  1.50 (0.89–2.52) \| \| Income quintile  1  2  3  4  5 \| 0.72 (0.48–1.07)  0.93 (0.64–1.36)  1.22 (0.84–1.75)  1.23 (0.84–1.79)  1 (ref) \| 0.98 (0.65–1.48)  1.10 (0.73–1.65)  1.37 (0.93–2.02)  1.17 (0.78–1.73)  1 (ref) \| 0.74 (0.51–1.07)  0.80 (0.56–1.13)  1.30 (0.90–1.86)  1.04 (0.73–1.48)  1 (ref \| \| Residence  Rural  Urban \| 0.75 (0.52–1.08)  1 (ref) \| 0.64 (0.44–0.93)  1 (ref) \| 0.77 (0.55–1.08)  1 (ref) \| \| Year of diagnosis  2007–2009  2010–2012  2013–2014 \| 1 (ref)  1.09 (0.80–1.49)  1.00 (0.72–1.38) \| 1 (ref)  0.96 (0.70–1.31)  0.94 (0.67–1.31) \| 1 (ref)  0.94 (0.71–1.26)  0.85 (0.63–1.14) \| |
| **Chau et al., (2019)** | **Relationship between Performance Status and QoL**  *Odds ratio (OR) by unit change from baseline in QLQ-C30 at Week 6 for ECOG Performance Status Group:*   \| QLQ-C30 Scale \| 5-point  OR (95% CI) \| 10-point  OR (95% CI) \| 15-point  OR (95% CI) \| 20-point  OR (95% CI) \| \| --- \| --- \| --- \| --- \| --- \| \| Physical Functioning      Emotional functioning      Role functioning      Cognitive functioning      Social functioning      Global QoL \| 0.83 (0.78-0.88)  0.90 (0.85-0.95)  0.91 (0.87-0.95)  0.93 (0.88-0.99)  0.94 (0.89-0.98)  0.90 (0.85-0.95) \| 0.69 (0.60-0.78)  0.80 (0.72-0.90)  0.83 (0.76-0.90)  0.87 (0.80-0.96)  0.81 (0.72-0.90)  0.81 (0.72-0.90) \| 0.57 (0.47-0.69)  0.72 (0.61-0.85)  0.75 (0.66-0.86)  0.81 (0.67- 0.98)  0.82 (0.71-0.94)  0.72 (0.61-0.86) \| 0.47 (0.36-0.61)  0.64 (0.51-0.81)  0.69 (0.58-0.82)  0.76 (0.59-0.97)  0.76 (0.64-0.92)  0.65 (0.52-0.81) \| |
| **Koh et al., (2014)** | **Relationship between BDNF Val66Met polymorphism and Mental Adjustment to Cancer:**  *Correlation coefficients for the difference in Mini-MAC sub-scales between the Met allele carriers’ group and Val homozygote group*   - Genotype of the BDNF Val66Met polymorphism x coping style (anxious preoccupation): r = 0.23 - Genotype of the BDNF Val66Met polymorphism x coping style (Helplessness/Hopelessness): r = -0.07 - Genotype of the BDNF Val66Met polymorphism x coping style (Fatalism): r = 0.07 - Genotype of the BDNF Val66Met polymorphism x coping style (Fighting spirit) r = -0.07 - Genotype of the BDNF Val66Met polymorphism x coping style (Cognitive Avoidance) r = -0.17   *Note:* These correlation coefficients were calculated using the means and SDs, based on an Effect Size Calculator (<https://www.campbellcollaboration.org/escalc/html/EffectSizeCalculator-R3.php>) |
| **Merchant et al (2021)** | **Relationship between patient characteristics and depression/anxiety**  *Odds of reporting moderate-severe symptoms (≥4–10) in depression/anxiety*   \| ***Covariate*** \| ***Depression or Anxiety*** \| \| --- \| --- \| \|  \| ***OR (95% CI)*** \| \| Sex      Male      Female \| 1 (ref)  1.58 (1.21–2.08) \| \| Age     ≤40     41-50     51-60     61-70     ≥71 \| 1.11 (0.59–2.11)  1.29 (0.84 - 2.02)  1.21 (0.88 - 1.66)  1.21 (0.89 - 1.64)  1 (ref) \| \| SES Quintile     1     2     3     4     5 \| 0.97 (0.64 - 1.48)  1.14 (0.77 - 1.69)  0.93 (0.64 - 1.35)  1.16 (0.79 - 1.71)  1 (ref) \| \| Cancer Site     Oesophageal     Gastric \| 1.18 (0.90 - 1.55)  1 (ref) \| \| Charlson Comorbidity index     0     1     2 and more \| 1.10 (0.79 - 1.53)  1.14 (0.78 - 1.67)  1 (ref) \| \| Distance to Nearest Hospital \|  \| \| < 10     11 - 50     > 50 \| 0.84 (0.62 - 1.14)  1 (ref)  0.81 (0.32 - 2.06) \| \| Patient Location  Rural  Urban \| 1 (ref)  1.37 (0.95 - 2.00) \| \| Region     Central East     Central West     East     Northeast     Northwest     South     Southeast     Southwest \| 1 (ref)  1.02 (0.70 - 1.50)  0.89 (0.60 - 1.33)  0.73 (0.44 - 1.23)  1.43 (0.66 - 3.39)  0.79 (0.44 - 1.43)  0.78 (0.47 - 1.29)  0.96 (0.66 - 1.39) \| |
